# Development of an efficient and precise adenine base editor (ABE) with expanded target range in allotetraploid cotton (*Gossypium hirsutum*)

**DOI:** 10.1186/s12915-022-01232-3

**Published:** 2022-02-15

**Authors:** Guanying Wang, Zhongping Xu, Fuqiu Wang, Yuefan Huang, Yanfeng Xin, Sijia Liang, Bo Li, Huan Si, Lin Sun, Qiongqiong Wang, Xiao Ding, Xiangqian Zhu, Luo Chen, Lu Yu, Keith Lindsey, Xianlong Zhang, Shuangxia Jin

**Affiliations:** 1 National Key Laboratory of Crop Genetic Improvement, Huazhong Agricultural University, Wuhan, Hubei 430070 People’s Republic of China; 2 Xinjiang Key Laboratory of Crop Biotechnology, Institute of Nuclear and Biological Technology, Xinjiang Academy of Agricultural Sciences, Wulumuqi, Xinjaing 830000 People’s Republic of China; 3 Department of Biosciences, Durham University, Durham, DH1 3LE UK

**Keywords:** Cotton, CRISPR/nCas9, dCas9, dCpf1, Adenine base editors (ABEs), Off-target mutations

## Abstract

**Background:**

Base editors (BEs) display diverse applications in a variety of plant species such as Arabidopsis, rice, wheat, maize, soybean, and cotton, where they have been used to mediate precise base pair conversions without the collateral generation of undesirable double-stranded breaks (DSB). Studies of single-nucleotide polymorphisms (SNPs) underpinning plant traits are still challenging, particularly in polyploidy species where such SNPs are present in multiple copies, and simultaneous modification of all alleles would be required for functional analysis. Allotetraploid cotton has a number of homoeologous gene pairs located in the A and D sub-genomes with considerable SNPs, and it is desirable to develop adenine base editors (ABEs) for efficient and precise A-to-G single-base editing without DSB in such complex genome.

**Results:**

We established various ABE vectors based on different engineered adenosine deaminase (TadA) proteins fused to Cas9 variants (dCas9, nCas9), enabling efficient A to G editing up to 64% efficiency on-target sites of the allotetraploid cotton genome. Comprehensive analysis showed that GhABE7.10n exhibited the highest editing efficiency, with the main editing sites specifically located at the position A5 (counting the PAM as positions 21–23). Furthermore, DNA and RNA off-target analysis of cotton plants edited with GhABE7.10n and GhABE7.10d by whole genome and whole-transcriptome sequencing revealed no DNA off-target mutations, while very low-level RNA off-target mutations were detected. A new base editor, namely GhABE7.10dCpf1 (7.10TadA + dCpf1), that recognizes a T-rich PAM, was developed for the first time. Targeted A-to-G substitutions generated a single amino acid change in the cotton phosphatidyl ethanolamine-binding protein (*GhPEBP*), leading to a compact cotton plant architecture, an ideotype for mechanized harvesting of modern cotton production.

**Conclusions:**

Our data illustrate the robustness of adenine base editing in plant species with complex genomes, which provides efficient and precise toolkit for cotton functional genomics and precise molecular breeding.

**Supplementary Information:**

The online version contains supplementary material available at 10.1186/s12915-022-01232-3.

## Background

The clustered regularly interspaced short palindromic repeat (CRISPR)/CRISPR-associated protein (Cas) system is the most powerful and widely adopted gene editing tool for research in life science [1]. This system causes DNA double-stranded breakage (DSB) in a site-specific manner and then leads to insertions and deletions (Indels) at the target sites by an endogenous repair mechanism, including high-fidelity homologous recombination (HR) and error-prone non-homologous end joining (NHEJ) repair pathway [2–6]. Many previous reports confirmed that a considerable number of important genetic diseases in humans, or agricultural elite traits of crops/livestock, are caused by single or a few base mutations [7]. However, the methods for inducing single-nucleotide changes by traditional chemical mutagens or homology-directed DSB repair (HDR) using template donor DNA are still technically challenging and may produce some unwanted mutations [2, 8]. Base editing based on CRISPR/Cas9 system is a promising precise point mutation technology without inducing DSBs at the target genomic locus. It normally uses a Cas9 variant (nCas9 or dCas9) and cytosine deaminase or adenine deaminase that was evolved artificially to perform precise single-base editing of target sites without DSBs, enabling the replacement of C by T or A by G [2, 9, 10].

Base editors (BEs) have been applied in various plant species such as Arabidopsis, rice, wheat, maize, soy bean, oilseed rape, and cotton [7, 11–13]. CBEs mainly mediate C-G to T-A base pair conversion in the editing window [14, 15]. Cytosine deaminases used in CBEs include human APOBEC3G, rat APOBEC1, human activation-induced cytidine deaminase (AID), and lamprey CDA1 [9, 16–19]. Recently, however, it has been found CBEs that uses rat APOBEC1 as the cytosine deaminase can cause unpredictable off-target mutations in rice and mouse embryo genomes [20–22]. ABEs deaminate adenine (A) to form hypoxanthine (I) that is replaced by guanine (G) in subsequent DNA repair and replication, which completes the transition of A to G by using the adenosine deaminase (TadA) from *Escherichia coli* fused at the N-terminus of nCas9 or dCas9. Through several rounds of TadA protein artificial evolution, a series of ABEs have been developed from in vitro experiments, of which ABE7.10 exhibits the highest efficiency and mediates an A-G transition at positions 4–7, while the editing windows of ABE6.3, ABE7.8, and ABE7.9 are at positions 4–9 [10, 23, 24]. However, n/dCas9 can only recognize the “NGG” protospacer-adjacent motif (PAM) sites. The limited PAM selection significantly hinders the design of sgRNA, and further applications of ABE and CBE vectors. Cpf1 (Cas12a) is another type of Cas protein and differs from Cas9 which recognizes T-rich PAM sequence (TTTV) at the target genome region [25]. Currently, catalytically inactive Cpf1 (dCpf1) together with cytosine deaminase (rat APOBEC1) has successfully been used to achieve cytosine base editing in animals [15, 26], thus substantially increasing the selection of target for base editing. However, fusion of dCpf1 and adenine deaminase to broaden the scopes of ABE targets has not been achieved in plants yet. In recent years, ABEs have been tested in model plant species and most of them are diploid species [3, 4, 27, 28]. The applications of ABEs in polyploids are still very limited, because the complex genomic structure of polyploid species required all alleles to be edited simultaneously in order to obtain the desired phenotypic traits [29].

The widely cultivated cotton species upland cotton (*Gossypium hirsutum*) is a global cash crop, for the production of both natural textile fiber and seed oil. Upland cotton is allotetraploid (A_t_D_t_) species with complex and large genome of 2.5 Gb with 52 chromosomes [30]. Advances in genome sequencing and the application of CRISPR/Cas9 and CRISPR/Cpf1 systems in cotton have greatly facilitated functional genomics research in cotton [25, 29–38]. As described previously, allotetraploid cotton has a number of homoeologous gene pairs with few SNPs between them across the A and D sub-genomes, and Allotetraploid cotton has a number of homoeologous gene pairs located in the A and D sub-genomes with few SNPs. However, the traditional CRISPR/Cas9 system is unable (or has low efficiency) to change these SNPs. In this situation, base editing, with its higher specificity in targeting specific A or C, has tremendous potential for the exploration of gene function in polyploid genomes, opening new opportunities for application in molecular breeding.

The *CHLOROPLASTOS ALTERADOS 1* (*CLA1*) gene encodes 1-deoxyxylulose-5-phosphate synthase, which is involved in the development of chloroplasts. Since mutation of the *CLA1* gene results in a distinct albino phenotype, it can be used as an easily recognizable marker for genome editing experiments [29, 39]. The phosphatidyl ethanolamine-binding protein (PEBP) family is involved in plant shoot architecture and flowering. The branches of cotton plants are either indeterminate or determinate. Nulliplex branch is typical of the determinate branch type and produces bolls that are borne directly on the main stem or on only one short fruit node. A clustered boll is formed on the top of the short fruit, producing a compact plant architecture suitable for mechanized harvesting, as occurs in USA, Brazil, Australia, and China. Previously, it has been reported following next-generation sequencing and bulked segregant analysis that several SNPs in the *GhPEBP* gene are associated with axillary flowering and/or clustered bolls. However, the relationship between these point mutations and phenotype could not be verified by traditional CRISPR / Cas9 technology [40]. Therefore, it would be valuable to investigate the use of a single-base mutation to generate an ideotype by manipulating *GhPEBP* gene in cotton [12, 40, 41].

In this report, eight adenine base editing vectors, based on the dCas9 or nCas9 system, were constructed for allotetraploid cotton. In addition, we also developed a dCpf1-based ABE vector and tested its efficacy in plants for the first time. The data illustrate the robustness of adenine base editing in plant species with a complex genome and provides a useful strategy for boosting base editing efficacy in plants.

## Results

### Determination of transgenes in the T0 cotton plants

**Fig. 1:**
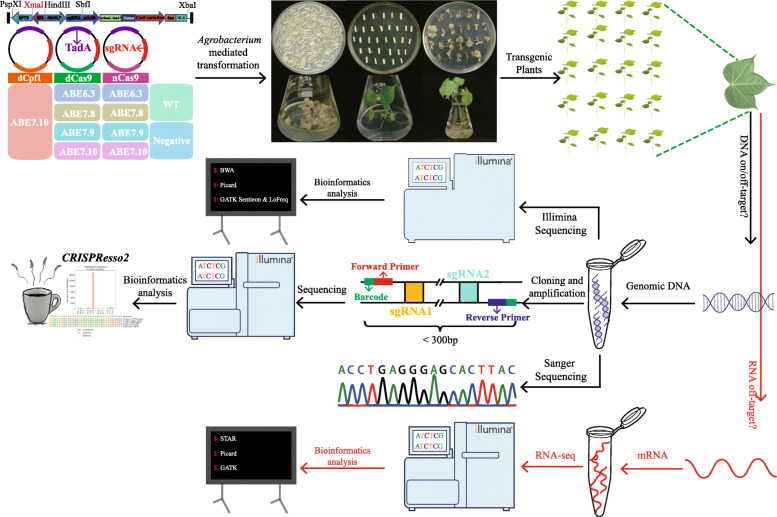
The vectors, workflow of GhABEs-mediated base edit in cotton. TadA, Escherichia coli TadA; sgRNA, small guide RNA; dCpf1, catalytically inactive L. bacterium Cpf1; dCas9, catalytically dead Cas9; nCas9, Cas9 nickase; ABE7.10, ABE6.3, ABE7.8, ABE7.9, four late-stage evolved adenosine deaminases

In order to test ABE activities in cotton plants, eight different ABE binary vectors were constructed for *Agrobacterium*-mediated transformation. The eight ABE vectors varied in the adenosine deaminase or Cas9 variants. Four different adenosine deaminases obtained from previously described ABEs (ABE6.3, 7.8, 7.9, and 7.10) were fused to nCas9 (D10A) or dCas9 (D10A, H840A) to generate eight ABE vectors. These eight ABEs, namely GhABE6.3n, GhABE6.3d, GhABE7.8n, GhABE7.8d, GhABE7.9n, GhABE7.9d, GhABE7.10n, and GhABE7.10d, were all codon-optimized based on cotton genomic preference for high expression level in transgenic cotton. A novel dCpf1 protein was synthesized after codon optimization and then fused with adenine deaminase from the GhABE7.10n vector and was designated as GhABE7.10dCpf1 (Fig. 1, Additional file 1: Appendix S1 and Additional file 1: Fig S1).

**Fig. 2:**
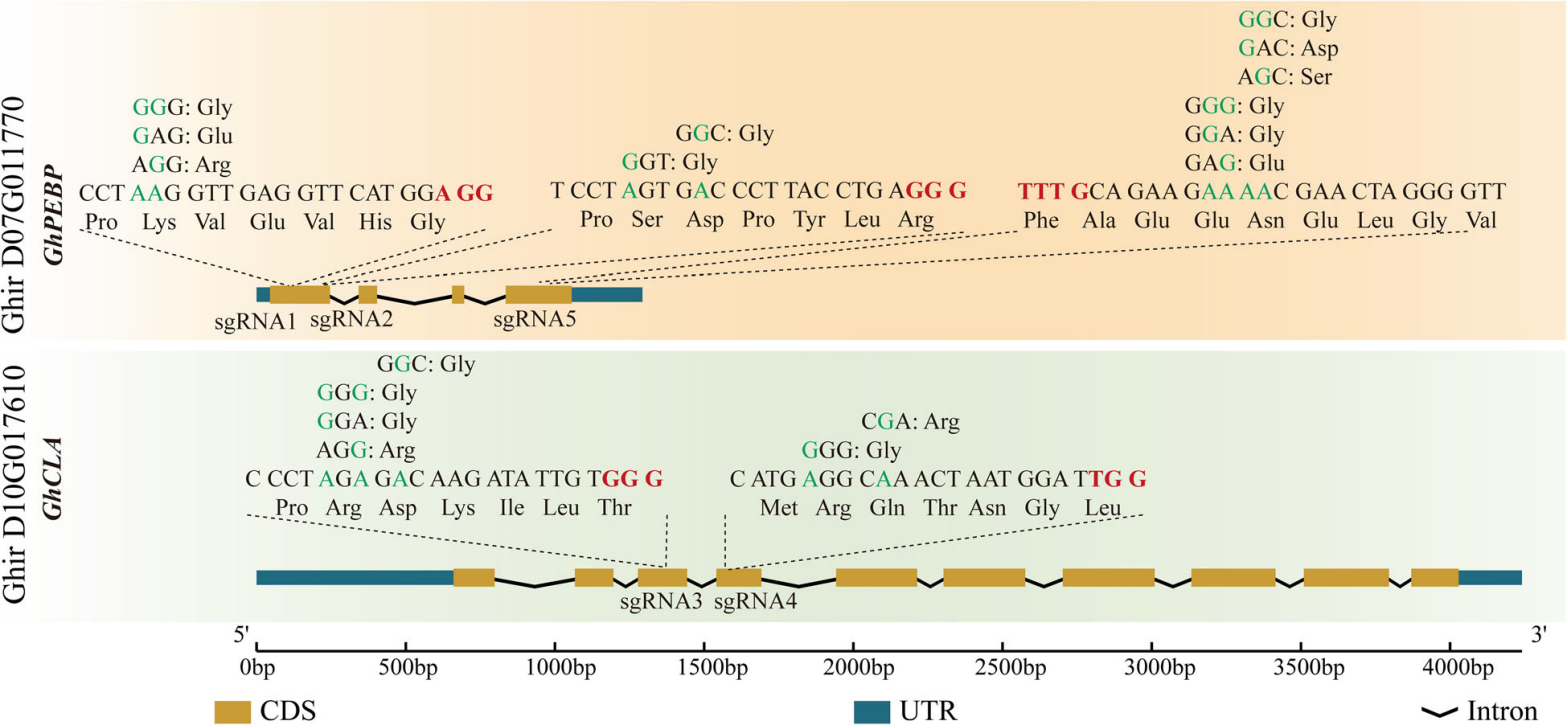
Details of the target sites for in GhABEs editing in *GhPEBP* and *GhCLA*. The illustrations show the expected amino acid change caused by the A-to-G conversion in *GhPEBP* and *GhCLA*. All the adenosines within the predictive windows in target sequences are highlighted in green, and the PAM sites are highlighted in red. The changes of amino acid sequences corresponding to nucleotides before and after GhABE editing are marked above the sequence

To evaluate the editing efficiency and the editing profiles of these ABEs in cotton, the *GhCLA* gene was selected as a marker gene and *GhPEBP* as a functional gene. Two pairs of sgRNAs for each of *GhPEBP* (tRNA-sgRNA1-tRNA-sgRNA2) and *GhCLA* (tRNA-sgRNA3-tRNA-sgRNA4) were designed (Fig. 2), targeting the adenine sites of these two genes. The two tRNA-sgRNA units of *GhPEBP* were cloned into the binary vectors GhABE6.3n, GhABE6.3d, GhABE7.8n, GhABE7.8d, GhABE7.9n, GhABE7.9d, GhABE7.10n, and GhABE7.10d. The two tRNA-sgRNA units of *GhCLA* were cloned into the binary vectors GhABE6.3n, GhABE7.8n, GhABE7.9n, and GhABE7.10n. Another sgRNA (sgRNA5) that targeting to *GhPEBP* was cloned into the binary vector GhABE7.10dCpf1. All these tRNA-sgRNA units were placed under the transcriptional control of the cotton endogenous U6 promoter according to our previous publication [29] (Fig. 1). Through *Agrobacterium*-mediated transformation and somatic embryogenesis, more than 200 independent transgenic T0 plants were obtained for further analysis, among which 62, 18, 21, 7, 13, 21, 10, 18, and 16 transformants were generated harboring T-DNA insertions of GhABE7.10n, GhABE7.10d, GhABE7.9n, GhABE7.9d, GhABE7.8n, GhABE7.8d, GhABE6.3n GhABE6.3d, and GhABE7.10dCpf1 vectors with sgRNAs targeting *GhPEBP*, respectively. In addition, 27, 18, 9, and 10 transformants were generated for GhABE7.10n, GhABE7.9n, GhABE7.8n, and GhABE6.3n vectors with sgRNAs targeting *GhCLA* (Table 1).

**Table 1:** Summary of editing frequencies of GhABE-n/dCas9 and GhABE-dCpf1

Base Editor	Adenine deaminase type	Cas9	Gene target	Number of checks	T0 positive strains	T1 positive strain	T0 editing efficiency (individual)
GhABE7.10n	TadA7.10	nCas9	*GhPEBP*	80	62	13	2–64%
GhABE7.9n	TadA7.9	nCas9	*GhPEBP*	24	21		3.83–6.1%
GhABE7.8n	TadA7.8	nCas9	*GhPEBP*	19	13		2.26–5.5%
GhABE6.3n	TadA6.3	nCas9	*GhPEBP*	15	10		3.12–4.89%
GhABE7.10n	TadA7.10	nCas9	*GhCLA*	42	27	6	~ 12.57%
GhABE7.9n	TadA7.9	nCas9	*GhCLA*	22	18		~ 3.77%
GhABE7.8n	TadA7.8	nCas9	*GhCLA*	11	9		~ 1.06%
GhABE6.3n	TadA6.3	nCas9	*GhCLA*	13	10		~ 1.00%
GhABE7.10d	TadA7.10	dCas9	*GhPEBP*	22	18	1	1.3–7.3%
GhABE7.9d	TadA7.9	dCas9	*GhPEBP*	9	7		1.06–3.46%
GhABE7.8d	TadA7.8	dCas9	*GhPEBP*	25	21		~ 3.5%
GhABE6.3d	TadA6.3	dCas9	*GhPEBP*	18	18		~ 3.44%
GhABE7.10dCpf1	TadA7.10	dCpf1	*GhPEBP*	18	16		~ 0.45

Note: Base editing efficiency was calculated by the ratio of reads with editing in the total reads at the target region of edited plants. Comparisons are based on base editing at sgRNA2- and sgRNA5-target sites

### Detection of on-target mutations by Sanger and target deep sequencing

**Fig. 3:**
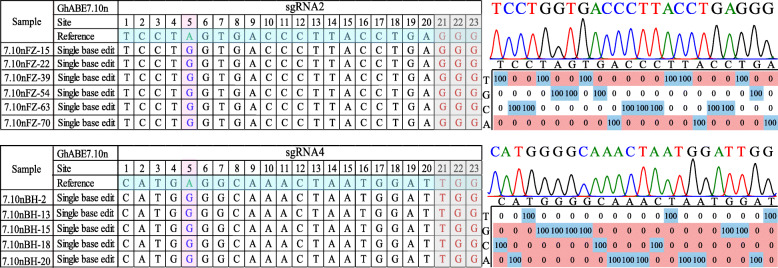
Representative editing profile from GhABE7.10n-edited plants for *GhPEBP* and *GhCLA* genes. The reference sequence in WT, edit site, and PAM are highlighted in cyan, violet, and grey

**Fig. 4:**
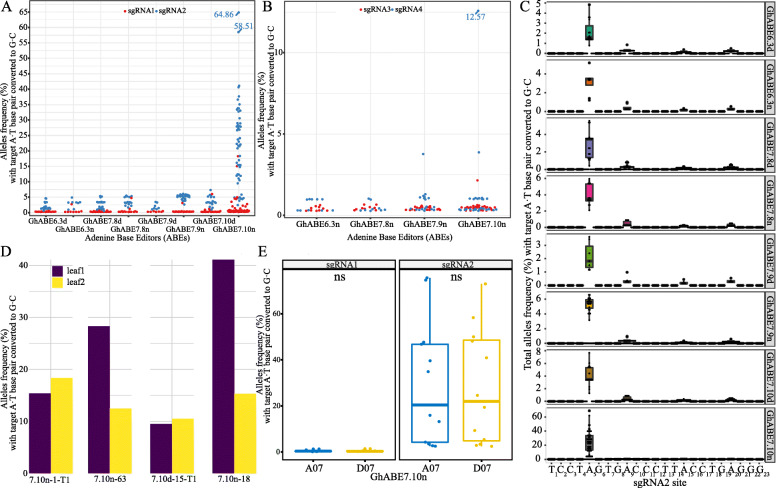
Identification of on-target mutations at *GhPEBP* and *GhCLA* target sites by targeted deep sequencing. **A** Base editing efficiency of all A-to-G conversion within sgRNA1 and sgRNA2 target region using eight GhABEs constructs revealed by deep sequencing for *GhPEBP* plants. Each point represents the editing efficiency of an independent sample. **B** Base editing efficiency of all A-to-G conversion within sgRNA3 and sgRNA4 target region using four GhABEs constructs revealed by deep sequencing for *GhCLA* plants. Each point represents the editing efficiency of an independent sample. **C** A•T to G•C base editing efficiencies of eight GhABEs at the target sgRNA2**.** All data were obtained from deep sequencing. **D** Comparison of A-to-G conversion efficiencies of different leaves in the same plant at sgRNA2 targets. Two leaves were detected for each single plant, and a total of four single plants were detected. **E** Comparison of the editing of GhABE7.10n on A and D subgenomes of allotetraploid cotton. Percentage of reads with target A•T to G•C substitution in total reads at sgRNA1 and sgRNA2 at target sites in At (blue dot) and Dt subgenomes (red dot) of cotton. Two-sided unpaired *t*-test

The sgRNA target regions of *GhPEBP* and *GhCLA* were individually amplified by PCR using specific primers with barcode tags (Additional file 1: Table S3 and S4), and the PCR products were selected for Sanger sequencing. The Sanger sequencing results revealed that every plant harbored at least one A to G substitution (with T-C conversions on the opposite strand) (Fig. 3). Sanger sequencing has high accuracy but its disadvantages of high sequencing cost and low throughput seriously limits large-scale application, whereas Illumina sequencing has several advantages over the Sanger sequencing technology: high throughput, high sensitivity, and low cost. Therefore, high-throughput deep sequencing (~ 10 million reads per locus) was also applied to analyze the A to G base editing profiles for all the edited plants in this report. Through targeted deep sequencing for more than 200 independent T0 plants, most plants were identified as harboring significant levels of A to G base substitutions at the sgRNA2 target site of *GhPEBP*. These data revealed that the editing efficiency of the sgRNA targeting at the 3′ end of genes (sgRNA2 of *GhPEBP*, sgRNA4 of *GhCLA*) is much higher than that of the sgRNA at the 5′end of the genes (sgRNA1 of *GhPEBP*, sgRNA3 of *GhCLA*) (Fig. 4A, B). Among these tested plants, the editing efficiency of sgRNA targeting the 5′ end of the genes is almost lower than 1%, so we subsequently focused on analyzing the base editing at the 3′ end of the target genes.

The efficiency of A to G substitution in the editing window of two sgRNAs target sites (sgRNA2 and sgRNA4) ranged from 0.27 to 64.9% (edited reads / the total sequenced reads) (Fig. 4A, B). To assess the biased sites of A-to-G transitions in the sgRNA editing window, the editing rate of each A-to-G transition was recorded and the results showed that the highest average editing efficiency was recorded at sgRNA2 target site. Among the tested eight different GhABE vectors, we found that the editing efficiency of the GhABE7.10n was much higher than the other seven vectors (Table 1, Fig. 4A–C). From the sequencing data of 62 GhABE7.10n-edited plants, A-to-G transition efficiency at position 5 of sgRNA2 target sites ranged from 5 to 64.9% (Fig. 4C). The second most successful vector is GhABE7.9n, with an editing efficiency of 4.3 to 6.1% at position 5 of the target site (Fig. 4C). The editing efficiency of the other GhABE vectors was lower than that of GhABE7.10n, ranging from 0.98 to 7.3% (Table 1, Fig. 4C). These deep sequencing data also showed that adenosine deaminase with dCas9 is not as efficient as adenosine deaminase with nCas9 in cotton (Fig. 4A–C), which is consistent with what was observed in base editing of human cells [9, 10].

It is noteworthy that we did not detect obvious A-to-G mutation at position 9, 15, or 20 in the sgRNA2 target sites of *GhPEBP* (Fig. 4C). By analyzing the editing window of all editors for *GhPEBP* and *GhCLA* genes, the frequency of mutation at A5 is significantly higher than at other adenine sites at the 20-bp sgRNA2 target sites (Fig. 4C, Additional file 1: Fig S2), particularly for the GhABE7.10n system. These data indicated that the GhABE7.10n vector performed accurate, effective, and clean single-base editing within the editing window of sgRNA target sites.

To further compare the editing efficiency of GhABE6.3n, GhABE7.8n, GhABE7.9n, and GhABE7.10n, we also designed sgRNAs (tRNA-sgRNA3-tRNA-sgRNA4) to target *GhCLA*. The result revealed lower A-to-G mutations at sgRNA3 target sites, whereas, at sgRNA4 target sites, a slightly higher mutation ratio was observed ranging from 0.96 to 18.3%. It also showed that GhABE7.10n was more effective than the other three base editors (Fig. 4B). The main difference between GhABE7.10n and GhABE6.3n, GhABE7.8n, GhABE7.9n was the amino acid sequence of the adenine deaminase (TadA). The differences between these amino acid sequences improve the compatibility with the deoxyadenosine substrate and broaden target sequence compatibility, resulting in the enhancement of deaminase activity and improvement of editing efficiency [10]. It is interesting to see that the A-to-G editing efficiency of different leaves from the same plants were divergent, which suggested the occurrence of chimeras might be widespread in cotton plants (Fig. 4D).

The ABEs have been applied in several diploid plant species including rice and Arabidopsis. The application of ABE in a complex genome like upland cotton (an allotetraploid species) has not been explored yet. Since there are At and Dt subgenomes in upland cotton, several sgRNAs were designed for *GhPEBP* to target homologous genes scattered in the subgenomes (Additional file 1: Fig S3A). We then analyzed target site editing efficiency from 62 T0 plants generated by the most efficient GhABE7.10n vector. The data showed that there was no obvious bias in editing efficiency between At and Dt subgenome (Fig. 4E). The mutation ratio at the target sites of the At subgenome ranged from 10 to 60% and 10 to 54% in the Dt subgenome. We also designed common primers based on the variation of DNA sequences between At and Dt subgenomes to perform PCR amplification of sgRNA2 sites. We were then able to distinguish the At and Dt subgenome sequences based on SNPs and InDels between each other. Sanger sequencing showed that A-to-G editing in both At and Dt subgenomes occurred at a similar level (Additional file 1: Fig S3B).

### The development of a new ABE system, GhABE7.10dCpf1, for cotton genome editing

In order to expand the target range (PAM sites) of the ABEs in cotton, a new ABE system, namely GhABE7.10dCpf1, was also developed, which is the fusion of dCpf1 (deactivated Cpf1) protein and adenine deaminase from the GhABE7.10 vector, and successfully applied in plants for the first time. We designed sgRNAs (sgRNA5) that targeted the *GhPEBP* gene (Fig. 2). Based on the deep sequencing data, anticipated base editing were confirmed at the target sites of sgRNA5 and the editing efficiencies ranged from 0.2 to 0.5% (Table 1). Previous studies in animal cells have shown that the editing window of dCpf1-CBE ranges from positions 8 to 13, counting the base next to PAM (TTTV) as position 1 [15]. The sequencing data revealed that the editing window in cotton plants spanned from positions 2 to 14 (Additional file 1: Fig S4). This low editing efficiency may be related to selecting bases adjacent to the A site at the target regions. Indeed, there are several adenines linked in the protospacer of the sgRNA5 (A2G3A4A5G6A7A8A9A10). Previous data in this report showed that nCas9-ABE exhibited higher editing frequency at the T4-A5 site than at A4–A5. Therefore, more studies are needed to determine the target preference of ABE7.10-dCpf1 in plants, which may help to increase its efficiency in the future [26].

### Whole genome sequencing analysis for the off-target effects in GhABE7.10-n/dCas9-edited cotton

**Fig. 5:**
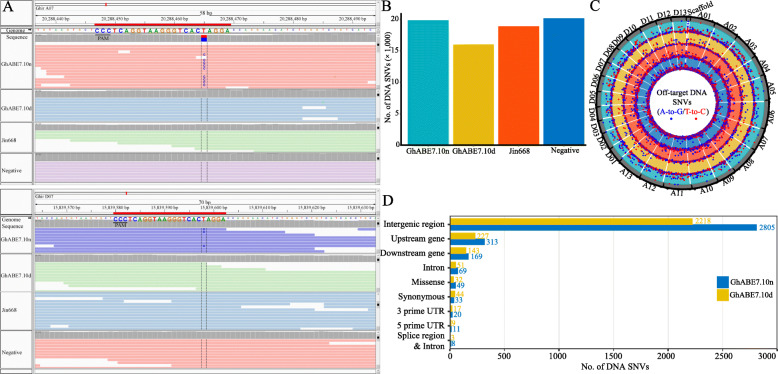
Genome-wide analysis of DNA off-target effect for the GhABE7.10 system by whole-genome sequencing. **A** Sequence alignment of sgRNA2 target sites on At and Dt subgenomes with IGV. The A>G (T>C) mutations edited by GhABE7.10d was detected at the A5 site of target region. The target sgRNA sequences are reverse complementarity and highlighted in different colors. **B** Numbers of total SNVs identified in the GhABE7.10n, GhABE7.10d, WT, and negative plants. **C** Characterization of DNA off-target SNVs (A-to-G/T-to-C) in GhABE7.10n and GhABE7.10d-edited plants. The DNA off-target SNVs (A-to-G/T-to-C) are randomly distributed across the cotton chromosomes in GhABE7.10n and GhABE7.10d plants. The tracks from outer to inner circles indicate the A-to-G (blue circle) and T-to-C (red circle) SNVs that identified at DNA levels of samples GhABE7.10n, GhABE7.10d, Jin668, and Negative. **D** Annotation of SNVs in the intergenic, exonic, intronic, upstream, and downstream regions of two GhABE7.10-edited T0 plants

To investigate the genome editing specificity of ABEs in cotton, two edited plants generated by the GhABE7.10n and GhABE7.10d vectors were chose to determine the off-target mutations by whole genome sequencing (WGS; with 50× sequencing depth)—a negative (following tissue culture and plant generation but without T-DNA insertion) and a wild type (WT, Jin668) as controls. According to the WGS results, we validated on-target editing at sgRNA2 target site (both At and Dt subgenome) in GhABE7.10n-edited plants by Integrative Genomics Viewer (IGV) (Fig. 5A), which was consistent with our target deep sequencing data (Fig. 4A). Single-nucleotide variants (SNVs) identified by WGS in these two edited plants were compared with potential off-target mutations (578 and 213 off-target sites for the sgRNA1 and sgRNA2) predicted using the Cas-OFFinder software [42] (Additional file 1: Table S5, S6). None of the SNVs in these two edited plants matched with these predicted off-target sites (Additional file 1: Fig S5A). After removing on-target SNVs, 19,863, 16,021, 18,892, and 20,193 SNVs were identified in plants edited by GhABE7.10n and GhABE7.10d, negative, and WT control, respectively (Fig. 5B). After filtering out background mutations using information from negative and WT plants, we mapped the distribution of SNVs and found that, in GhABE7.10n and GhABE7.10d-edited plants, these SNVs exhibited an apparently random distribution on the chromosomes and no mutation hotspots were detected (Fig. 5C). In addition, these A-G and T-C SNVs were concentrated in intergenic regions of the genome, where there was no confirmed genetic information (Fig. 5D). We suggest that these random SNVs were derived from somaclonal and/or germline variations, which would not affect the target genes’ function, nor produce any unexpected phenotypes. In summary, these results revealed that the GhABE7.10 did not induce off-target mutations in cotton genome, presumably because TadA7.10 is derived from an engineered RNA adenosine deaminase with high fidelity. Previous studies have speculated that engineered RNA adenosine deaminase does not show excessive DNA base editing, thus avoiding the generation A-G SNVs outside the ABE editing windows [20].

### Off-target effects of GhABE7.10-n/dCas9 at RNA level

**Fig. 6:**
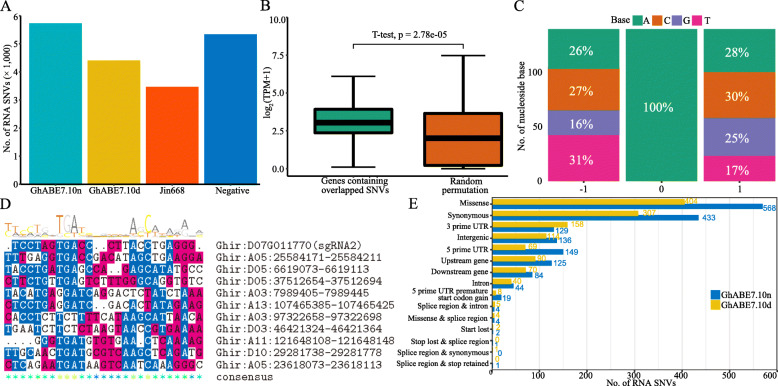
Characterization of off-target RNA-SNVs. **A** The number of A-to-G and U-to-C mutations for GhABE7.10n, GhABE7.10d, WT, and negative plants. **B** Expression of genes containing overlapping off-target RNA-SNVs and random simulated genes induced with GhABE7.10. Two-sided unpaired *t*-test. **C** Sequence derived from off-target RNA-SNVs of GhABE7.10. Analysis was performed on RNA-seq data using cDNA, and thus every T depicted should be considered a U in RNA. Three base sequence before and after the off-target A site explains whether the mutation site has base preference. **D** Similarity between adjacent sequences of off-target RNA-SNVs with sgRNA2 target sequences. The most similarity ten off-target SNVs (top 10) were shown. **E** The number of different locations of SNVs for two GhABE7.10-edited T0 plants

Several previous reports have revealed that the ABE7.10 system exhibits high-level off-target effects in animal cellular RNA [43]; however, any possible off-target RNA mutations induced by ABE7.10 have not been investigated in plants. To evaluate the extent of cellular RNA editing by GhABE7.10n and GhABE7.10d in cotton, we also chose the same four plants, used for DNA off-target analysis described previously, for RNA sequencing (RNA-seq) with an average 50× sequencing depth (Fig. 1). The RNA-SNVs identified in the two edited plants were compared with the off-target mutations predicted by using the Cas-OFFinder software [42] (Additional file 1: Table S5, S6). None of the RNA-SNVs detected in the two edited plants overlapped with the predicted off-target sites (Additional file 1: Fig S5B). Based on the RNA-SNVs identified from RNA-seq data, the number of RNA-SNVs found in GhABE7.10n is slightly higher than that in the negative and WT controls (Fig. 6A). After filtering out background mutations based on the sequencing data from negative and WT plants, the RNA-SNVs between samples GhABE7.10n and GhABE7.10d were compared, and there were 145 overlaps identified, which indicated these SNVs were not related to the differences in Cas9 variants or expression levels (Additional file 1: Table S7) and may be related to the natural action of adenine deaminase on RNA [43]. By comparing the expression levels of randomly selected genes from the transcriptome to genes containing RNA-SNVs identified in GhABE7.10-edited plants, these RNA-SNVs were found to be substantially enriched in genes with high transcription levels (Fig. 6B).

To investigate whether GhABE7.10 exhibited any base preference for nucleotide compositions near to the adenines of RNA-SNVs, we analyzed all the adjacent 3-bp sequences of the RNA-SNVs. The results showed a consensus motif TAM (M = A or C) in RNA-SNVs edited by the GhABE7.10 base editor (Fig. 6C). Additionally, 1540 sequences with PAM NGG (potential RNA off-target sites) were screened out in 20-base sequences containing RNA-SNVs and compared with the sgRNA2 target sequences. No similarity was found between them (Fig. 6D and Additional file 1: Fig S6), indicating these RNA-SNVs are unrelated to sgRNA target sites. The annotation of RNA-SNVs using SnpEff [44] revealed that they were located in both coding and non-coding sequences. The predominant RNA-SNVs are 568 missense mutations and 433 synonymous mutations (Fig. 6E).

In conclusion, there were no A-G SNVs detected at the predicted off-target sites in two edited plants. The number of RNA-SNVs in GhABE7.10n-edited plants was slightly higher than in the negative and WT controls. These low levels of RNA off-target editing were possibly caused by overexpression of TadA7.10, which is consistent with a previous report [43].

### The base edits produced by ABE are faithfully inherited from T0 parental plants to T1 progenies

**Fig. 7:**
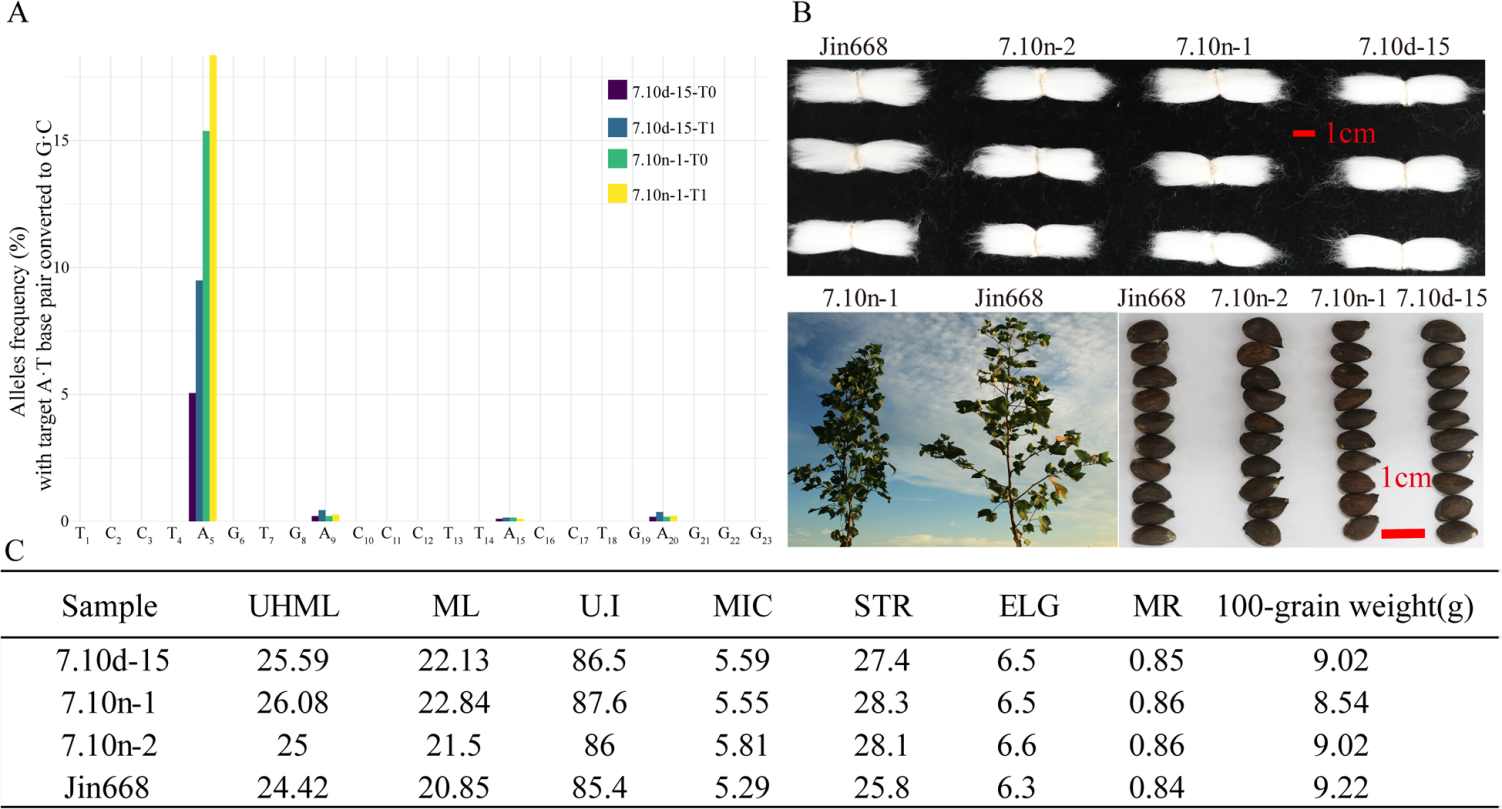
Inheritance and compact phenotype of progenies of *GhPEBP* gene edited by GhABE. **A** Representative targeted deep sequencing results from 7.10d-15 and 7.10n-1 cotton T1 seedlings. Genotyping of independent T0 plants and their T1 progeny at the sgRNA2 of *GhPEBP* gene. **B, C** The phenotypic and agronomic characters (fiber properties, 100-grain weight, and seed size) in a GhABE edited line. The *GhPEBP* edited line 7.10n-1 shows increased number of lateral branches or shortened length of fruit nodes. The red bar = 1 cm

From the data of the target deep sequencing and whole-genome resequencing of T0 plants, we can see that GhABE7.10n can work efficiently for the base editing in cotton. To test whether the A-to-G mutation in T0 plants could be inherited through the germline, T1 seeds harvested from 7.10n-1-T0 and 7.10d-15-T0 were sown, and T1 leaves were collected for positive identification and target deep sequencing analysis (Additional file 1: Table S2 and S3). The deep sequencing data show that single-base mutation at the A5 position occurred with a frequency of 18.35% and 9.49% in the 7.10n-1-T1 and 7.10d-15-T1 plants respectively, compared to 15.38% and 5.05% in the 7.10n-1-T0 and 7.10d-15-T0 (Fig. 7A and Additional file 1: Fig S7). Apparently, the base editing efficiency of T1 lines was higher than in the T0 parental plants (Fig. 7A), which indicates that some new editing events or more cells with the same editing were generated in the T1 plants. We also identified one transgene-free line 7.10n-1-T1-1 from 7.10n-1-T0, which showed 28% editing efficiency at target sgRNA site of the *GhPEBP* gene (Additional file 1: Table S2 and Fig S8). Importantly, these data confirmed that the mutations produced by ABEs can be faithfully inherited from T0 parental plants to T1 progenies. Moreover, several T1 plants with a higher editing ratio at the target gene-*GhPEBP* exhibited the desired compact phenotypes, i.e., with increased numbers of lateral branches and shortened fruit nodes (Fig. 7A, Additional file 1: Fig S9, S10). In order to assess the effect of single-base mutations on major agronomic traits of cotton, fiber properties and seed quality, fiber length, strength, and micronaire values and 100-grain weight were measured for the edited plants. The results show that there were no obvious changes in fiber and seed quality in these three edited lines compared with the WT (Fig. 7B, C).

In conclusion, GhABE7.10n can efficiently achieve A-to-G substitutions within editing windows in the cotton genome and these can be transmitted to the next generation, resulting in phenotypic changes without adverse side effects on major agronomic traits.

## Discussion

With the rapid development of life science, high-precision base editing technology has become an important new tool with possibilities for gene therapy of rare diseases and crop genetic improvement [45–48]. CBE-mediated C-T and ABE-mediated A-G base editing technologies have been widely used in plants, providing important technical support for studying plant gene function and the role of gene regulatory elements [2, 7]. Although CBE has been successfully used in allotetraploid cotton, it is necessary to expand new single-base editing tools to edit more nucleotide types in this species [12]. Here we describe the development of various cotton-compatible ABE vectors (GhABEs) with optimized adenosine deaminase and n/dCas9. Using these GhABEs, we achieved allelic editing of adenine to guanine efficiently and specifically in the allotetraploid genome of cotton. These functional GhABEs differ in adenine deaminase from four newly developed ABEs in human cells (ABE6.3, 7.8, 7.9, and 7.10) and variants of Cas9. Our results show that GhABE7.10n is the most efficient editing vector, and the editing produced by GhABE7.10n generated A-to-G conversion only within the editing window.

The editing window is essential for the applications of base editors, because it determines which site(s) can be targeted. GhABE7.10n has shown a narrow editing window, which will be essential for precision breeding. However, a relatively narrow editing window means fewer target nucleotides, making more difficult the design of sgRNAs because of the restricted PAM sequences, which in turn could restrict the use of ABE system in cotton or other allotetraploid plant species. Therefore, we need to continue to develop base editors for different editing windows and for identifying different types of PAMs.

Previous studies have speculated that the editing efficiency of deaminase with nCas9 is higher than that with dCas9, due to nCas9 being able to nick the non-edited strand, with a subset of this stretch of ssDNA in the R-loop serving as an efficient substrate for deaminase to effect direct, programmable base conversion in DNA. At the same time, the adenine deaminase TadA7.10 has the strongest editing activity [9, 10, 26]. Therefore, GhABE7.10n assembled from nCas9 and TadA7.10 showed the best editing efficiency compared with other combinations, such as dCas9 and Tad7.10.

It has been reported that the off-target effects of base editing are diverse across different species. ABE7.10n has been proven to generate high-frequency off-target RNA mutations and ABE7.10 ^F148A^ showed a low level of RNA off-target effects when TadA-7.10 was introduced a F148A mutation in animal cells, but has not been investigated in plant cells [43]. In addition, several T1 plants with target editing of *GhPEBP* exhibited phenotypic alterations. It has been reported that terminal flowers appeared after silencing of this *GhPEBP* gene by RNA interference (RNAi), resulting in a determinate architecture [49]. In the current report, we used GhABE7.10n to create point mutations of this gene which resulted in shorter internodes and more fruit branches. These differences in phenotype may be due to the gene’s functional redundancy in allotetraploid cotton, whereby the mutation of one copy of the target gene can be partially rescued by its allele in the other subgenome. In addition, the frequency of point mutation caused by GhABE7.10n is not 100% and chimeric mutants may generate an intermediate phenotype. The compact phenotype caused by GhABE7.10n between monopodial and sympodial branches provides new possibilities for high-density crop planting. In addition, the two T1 generation single plants 7.10n-1-T1 and 7.10n-1-T1-1 from 7.10n-1-T0 showed different editing efficiency. The 7.10n-1-T1 was positive by PCR amplifying of gRNA target sites and selecting maker gene *NPTII* DNA sequence and had an editing rate of 18.35%, while the 7.10n-1-T1-1 was nontransgenic due to the absence T-DNA insertion with the editing rate of up to 28.2%. According to Mendelian heritability, if a T1 cotton plant is considered to be transgene free, the theoretical ratio of an edited allele versus non-edited allele should be more than 25%. Therefore, 7.10n-1-T1-1 was considered as a transgene free and the mutation caused by *GhABE* can be stably inherited to the next generation. In order to provide more evidence for the heritability of mutation, the best way is to further detect the editing rate of T3 generation for 7.10n-1-T1-1. Unfortunately, till now, the T1 generation plants of 7.10n-1-T1-1 are still in the greenhouse. Given all of that, GhABE7.10n is an effective and precise tool that can accomplish site-specific A-to-G base editing and improve important agronomic traits in cotton.

The PAM sequence is essential for the wide uptake of base editing systems, as it determines the choice of targets [50]. Although many CRISPR/Cas9 variants, as well as Cpf1 with different PAM, have been developed and successfully used in the genomes of animals and plants [51], the base editing system, with its fusion of catalytically dead LbCpf1 (dCpf1) and deaminase, has not been used in plants. Previous studies have shown that the dLbCpf1-mediated CBE system can work in human cells [15]. Here we also established a dLbCpf1-mediated ABE tool with optimized dLbCpf1 and adenine deaminase TadA-7.10. To our knowledge, this is the first time that dLbCpf1-mediated base editing system has been used in plants. However, the new GhABE7.10dCpf1 system has low editing activities compared with GhABE7.10nCas9, probably because of the sgRNA sequence context preference of target A for adenine deaminase TadA-7.10 substrates. In addition, ABEs have shown limited compatibility with Cas homologs. Some homologs such as SaCas9, SaCas9-KKH, SpCas9-NG, and CP-Cas9s are compatible with ABEs, but editing efficiencies are substantially lower than those of the corresponding CBEs. Other homologs such as LbCas12a and enAsCas12a show virtually no activity as an ABE [26].

In summary, we have established a series of CRISPR-Cas9/Cpf1-based ABEs in cotton. GhABE7.10n can perform targeted A to G base editing with very low levels of RNA off-target and without DNA off-target. These tools should provide important technical support for cotton genome function analysis, crop genetic improvement, and the breeding of new varieties.

## Conclusions

Nine adenine base editing tools, based on dCas9, nCas9, and dCpf1, were used in cotton for the first time. Our results provide efficient and precise adenine single-base editing tools for cotton functional genomics and precise molecular breeding.

## Methods

### Plasmid construction

ABE (adenine base editor) plasmid vectors were modified from the *G. hirsutum*-Base Editor 3 (GhBE3) generated in our recent report [12]. Cytosine deaminase (APOBEC), nCas9, and UGI in GhBE3 were deleted by double digestion of PacI (NEB) and XbaI (NEB). In order to make adenine deaminase work efficiently in cotton, the coding regions of TadA-TadA6.3-dCas9, TadA-TadA6.3-nCas9, TadA-TadA7.8-dCas9, TadA-TadA7.8-nCas9, TadA-TadA7.9-dCas9, TadA-TadA7.9-nCas9, TadA-TadA7.10-dCas9, and TadA-TadA7.10-nCas9 were codon-optimized for expression in cotton and synthesized by GenScript (Nanjing, China) as described by Gaudelli et al. [10]. This eight synthetic nucleic acid sequence was inserted into the binary vector GhBE3 that had been digested by double enzymes to generate eight *G. hirsutum*-Adenine Base Editors (GhABEs), namely GhABE6.3nCas9, GhABE6.3dCas9, GhABE7.8nCas9, GhABE7.8dCas9, GhABE7.9nCas9, GhABE7.9dCas9, GhABE7.10nCas9, and GhABE7.10dCas9. The eight GhABEs were linearized by SbfI (NEB) and BstBI (NEB) double digestion to delete the sgRNA expression cassettes. A fragment including SbfI (NEB) and BstBI (NEB) sites and the sgRNA scaffold with two 20 bp-target sequences of *GhPEBP* (sgRNA1-sgRNA2) was synthesized by GenScript (Nanjing, China) and then cloned by PCR (Additional file 1: Table S1). The sgRNA expression cassettes with two 20 bp-target sequences of *GhPEBP* were transferred into the appropriate eight GhABEs using the ClonExpressII One Step Cloning Kit (Vazyme, Nanjing, China). For sgRNA of *GhCLA* target gene (sgRNA3-sgRNA4), only GhABE6.3nCas9, GhABE7.8nCas9, GhABE7.9nCas9, and GhABE7.10nCas9 were selected and transferred as described for the *GhPEBP* gene. The GhABE7.10dCpf1 vector was modified from GhABE7.10nCas9. The nucleotide sequence of dCpf1 was derived from the dLbCpf1-BEs vector [15]. After cotton codon optimization, dCpf1 was synthesized (the GenScript company, Nanjing, China) and cloned into the GhABE7.10nCas9 vector from which nCas9 was deleted by double digestion. The sgRNA expression cassettes with one 23 bp-target sequence of *GhPEBP* (sgRNA5) were designed by website and synthesized by GenScript (Nanjing, China), and transferred into GhABE7.10dCpf1 using the ClonExpressII One Step Cloning Kit (Vazyme, Nanjing, China).

### *Agrobacterium*-mediated cotton transformation

All the GhABEs plasmid vectors were transformed into Agrobacterium strain GV3101 (kanamycin as selectable marker) by electroporation, and Agrobacterium-mediated transformation of cotton cultivar J668 was performed according to previous publications [35, 52].

### Deep sequencing to detect target site mutations

Genomic DNA of transgenic cotton plants was isolated from T0 and T1 generation (for genetic identification of offspring) and control cotton plants using the CTAB method [53]. Specific primers (Additional file 1: Table S2) for nCas9 and sgRNA sequence were used to confirm transgenics. To track all sequencing data back to a single original transgenic plant/sample, each sample was designed with a pair of unique barcode tags consisting of six to seven bases [54], using our own Python script. Each pair of barcode tags was added to the 5′ end of the forward and reverse primers which amplify various target sites (Additional file 1: Table S3, S4). T-DNA insertions confirming transgenic plants were amplified by PCR using specific primers with barcode tags and the amplicons were mixed in equal amounts to construct a sample library. To remove polymerase, the library of PCR products was purified using PCR Purification Kit (OMEGA, D2500-02). Finally, the purified library was prepared with no PCR amplification (PCR-free) for Illumina sequencing library and sequenced on an Illumina HiSeq 2500 sequencer following the manufacturer’s protocol (Illumina, San Diego, CA). The raw data were filtered to remove low-quality reads and adapter under command parameters: LEADING:5 TRAILING:5 SLIDINGWINDOW:4:20 MINLEN:50 using Trimmomatic [55]. FastQC [56] quality visualization was applied, and clean reads would be used for further analysis. Demultiplexing was processing where reads from FASTQ sequencing files were assigned to each sample based on the barcode tags. CRISPResso2 [57] with parameters “--quantification_window_size 10 --quantification_window_center -10 --base_editor_output --conversion_nuc_from A --conversion_nuc_to G” was used to analysis of genome editing. In addition, wild-type (WT) plants were used to filter out background mutations in the cotton population. Control plants (Negative) were used to evaluate the mutations occurring during tissue culture and transformation.

### On-target mutation analysis by Sanger sequencing

For each confirmed transgenic plant, T-DNA insertions were amplified by PCR using specific primers (Additional file 1: Table S2). The PCR products were purified with an EasyPure PCR Purification Kit (TransGen Biotech, Beijing, China) and then ligated into the pGEMT-Easy vector using T4 DNA ligase (Promega, Madison, USA). The plasmid containing the amplicons was transformed into *E. coli* by heat shock. Positive monoclones were Sanger sequenced and quantified using EditR 1.0.9 (https://moriaritylab.shinyapps.io/editr_v10/).

### Detection of off-target mutations by WGS

Genomic DNA was extracted from young leaves of an individual cotton plant (transgenic, negative (undergone tissue culture and plant regeneration but without T-DNA insertion) and WT) using the Plant Genomic DNA Kit (Tiangen Biotech, China). A total of four plants, including one WT plant, one negative plant, and two base editor plants, edited by GhABE7.10-nCas9 and GhABE7.10-dCas9 with two pairs of sgRNAs for *GhPEBP* (tRNA-sgRNA1-tRNA-sgRNA2) gene, were used to evaluate genome-wide genetic variants. For each plant, ca. 1.5 μg genomic DNA was prepared to generate a standard Illumina short-read genomic library and paired-end sequencing (2 × 150 bp) on the Illumina HiSeq 2500 sequencer in accordance with the manufacturer’s recommendations (Illumina, San Diego, CA), ultimately resulting in more than 1 Tb raw reads (the average depth being 50×). The filtered (Trimmomatic [55]) and quality-checked (FastQC [56]) clean reads were mapped to the reference-grade *Gossypium hirsutum* L. acc. TM-1 genome [30] (http://cotton.hzau.edu.cn/EN/download.php) with BWA (v0.7.17) [58]. Samtools (v1.9) [59] was used to filter multiple mapping reads and sort BAM files by read name. The picard program (v2.1.1) (http://broadinstitute.github.io/picard/) was used to mark duplicative reads, and the Genome Analysis Toolkit (GATK v4.1) [60], Sentieon (201911) (https://www.sentieon.com/), and LoFreq (v2.1.5) [61] were employed to variant calling. The high-confidence SNVs, which had to be identified by all three software and filtered with parameters “QD < 2.0 || FS > 60.0 || MQ < 40.0 || MQRankSum < -12.5 || ReadPosRankSum < -8.0,” were used for subsequent analysis.

Off-target sites were predicted by Cas-OFFinder (v2.4) [42], allowing up to 5-nt mismatches. SnpEff [44] was used to annotation and predicts the effects of each off-target variant based on *Gossypium hirsutum* L. acc. TM-1 genome [30].

The Integrative Genomics Viewer (IGV) [62–64] was used to check obtained SNVs.

### Detection of off-target mutations in RNA sequence

The samples from individual plants that were used to detect off-target genomic mutations were also prepared for RNA-editing analysis. The total RNA of four plants described above was isolated as previously described [65]. For library construction, mRNAs were fragmented and converted to cDNA using oligo (dT) primers (Invitrogen, Carlsbad, CA, USA). High-throughput mRNA sequencing was carried out using the Illumina Hiseq platform according to the manufacturer’s recommended protocol. We generated an average of 50× paired-end reads for each sample. Illumina paired-end reads were processed as previously described. In brief, FastQC (v.0.11.8) and Trimmomatic (v.0.36) were used for quality control. Qualified reads were mapped to the reference genome *Gossypium hirsutum* L. acc. TM-1 genome [30] (http://cotton.hzau.edu.cn/EN/download.php) using STAR (v.2.7.1a) in two-pass mode. Picard tools (v.2.9.2) was then applied to sort and mark duplicates of the mapped BAM files. RNA base editing variants were called using GATK (v4.1) and Sentieon (201911) (https://www.sentieon.com/) from the refined BAM files. High-confidence SNVs were identified using both software. To identify variants with high confidence, we filtered variants with parameters “QD < 2.0 || FS > 60.0 || MQ < 40.0 || MQRankSum < -12.5 || ReadPosRankSum < -8.0” and clusters of at least five SNVs that were within a window of 35 bases. The sum of mutations A-to-G and T-to-C were counted as edited as previously described [43].

SnpEff [44] was also used to annotate and predict the effects of each off-target variant as for the above WGS analysis.

RSEM (v.1.3.3) was used to estimate the gene expression levels with default parameters, and gene abundances were quantified and presented as transcripts per million kilobases (TPM).

The 20-bp sequences adjacent to off-target RNA-SNVs (containing NGG PAM in downstream region) were extracted from the *Gossypium hirsutum* L. acc. TM-1 genome [30] and aligned using the R package msa [66].

### Comparisons of editing efficiency and accuracy of different ABEs in cotton

To determine the optimum editor for cotton, we performed a comprehensive comparison of efficiencies with introduced point mutations of A•T to G•C within the sgRNA target, point mutations (not A•T to G•C) within or flanking of the sgRNA target, and off-target mutations in all carriers. All statistical analyses were performed using R package 3.6.1 (http://www.R-project.org/). In the two-sided test, *P* < 0.05 was considered as being statistically significant. All plots were performed using R ggplot2 package, and final stage editing and composition of main and supplemental figures was done in Adobe Illustrator CS6.

## Supplementary Information

**Additional file 1: **Supplementary Figs. 1-10. **Figure S1.** Schematic representation of the base editors. **Figure S2.** The identification of on-target mutations a GhCLA target sites by targeted deep sequencing. Base-editing efficiency of all A to G conversion within sgRNA3 and sgRNA4 target region using four GhABEs constructs revealed by deep sequencing for single plant. **Figure S3.** The different of editing efficiency between At and Dt subgenomes o GhPEBP gene. (A) Multiple sequence alignment of sgRNA target regions for GhPEBE homologous genes, which shown the SNPs and InDels between At and Dt subgenomes. (B) Sanger sequencing of sgRNA2 in three lines. **Figure S4.** Allele compositions following treatment with GhABE7.10dCpf1 at the sgRNA5 of GhPEBP. **Figure S5.** Venn diagram analysis of the SNVs identified in ABE base editor together with the off-target sites predicted by Cas-OFFinder. (A) DNA SNVs from WGS data. (B) RNA SNVs from RNA-seq data. **Figure S6**. Similarity between adjacent sequences of off-target RNA SNVs with sgRNA1 target sequences. The most similarity ten off-target SNVs (top 10) were shown. **Figure S7**. The allele composition of T0 and T1 generation at sgRNA2 of GhPEBP was treated with GhABE7.10n or GhABE7.10d. **Figure S8.** The Illumina sequencing of transgene-free line isolated from T0 plants. (A) PCR detection of transgene-free plants. (B) The editing efficiency of the transgene-free plants detected by target deep sequencing. **Figure S9.** Comparison of the number of lateral branches and length of fruit nodes of a base-edited T1 plant generated via GhABE7.10n with wild-type Jin668 plant. Scale bar, 1 cm. **Figure S10**. The long-branching WT phenotype (right) and the GhPEBP phenotype (left) in upland cotton. Local area and local magnification are represented by dashed lines of different colors.

**Additional file 2: **Supplementary tables 1-7. **Table S1.** Primers used for vectors construction. **Table S2.** Primers used for positive test. **Table S3.** Barcode primers for detecting off-target in base editing T0 transgenic plants with deep sequencing for *GhPEBE* gene. **Table S4.** Barcode primers for detecting off-target in base editing T0 transgenic plants with deep sequencing for *GhCLA* gene. **Table S5.** Summary of genome-wide potential off-targets predictions by Cas-OFFinder tools for target sgRNA1. **Table S6.** Summary of genome-wide potential off-targets predictions by Cas-OFFinder tools for target sgRNA2. **Table S7.** Expression levels (TPM) of TadA, nCas9 and dCas9 in GhABE7.10 edited plant.

**Additional file 3:.** Supplementary Appendix 1: Sequences of each component of nine GhABEs system.

## Data Availability

All data generated or analyzed during this study are included in this published article, its supplementary information files and publicly available repositories. All the sequencing data have been deposited in the NCBI Sequence Read Archive (SRA) under project accession numbers PRJNA774486 and PRJNA774488.
