# Supplementary material for: Development of an efficient and precise adenine base editor (ABE) with expanded target range in allotetraploid cotton (Gossypium hirsutum)
Source: BMC Biol. 2022 Feb 15;20:45. doi: 10.1186/s12915-022-01232-3 (PMC8845244; doi:10.1186/s12915-022-01232-3)
Supplement: Supplementary file 1 — Additional file 1: Supplementary Figs. 1-10. Figure S1. Schematic representation of the base editors. Figure S2. The identification of on-target mutations a GhCLA target sites by targeted deep sequencing. Base-editing efficiency of all A to G conversion within sgRNA3 and sgRNA4 target region using four GhABEs constructs revealed by deep sequencing for single plant. Figure S3. The different of editing efficiency between At and Dt subgenomes o GhPEBP gene. (A) Multiple sequence alignment of sgRNA target regions for GhPEBE homologous genes, which shown the SNPs and InDels between At and Dt subgenomes. (B) Sanger sequencing of sgRNA2 in three lines. Figure S4. Allele compositions following treatment with GhABE7.10dCpf1 at the sgRNA5 of GhPEBP. Figure S5. Venn diagram analysis of the SNVs identified in ABE base editor together with the off-target sites predicted by Cas-OFFinder. (A) DNA SNVs from WGS data. (B) RNA SNVs from RNA-seq data. Figure S6. Similarity between adjacent sequences of off-target RNA SNVs with sgRNA1 target sequences. The most similarity ten off-target SNVs (top 10) were shown. Figure S7. The allele composition of T0 and T1 generation at sgRNA2 of GhPEBP was treated with GhABE7.10n or GhABE7.10d. Figure S8. The Illumina sequencing of transgene-free line isolated from T0 plants. (A) PCR detection of transgene-free plants. (B) The editing efficiency of the transgene-free plants detected by target deep sequencing. Figure S9. Comparison of the number of lateral branches and length of fruit nodes of a base-edited T1 plant generated via GhABE7.10n with wild-type Jin668 plant. Scale bar, 1 cm. Figure S10. The long-branching WT phenotype (right) and the GhPEBP phenotype (left) in upland cotton. Local area and local magnification are represented by dashed lines of different colors. [file 12915_2022_1232_MOESM1_ESM.pptx]

## Slide 1
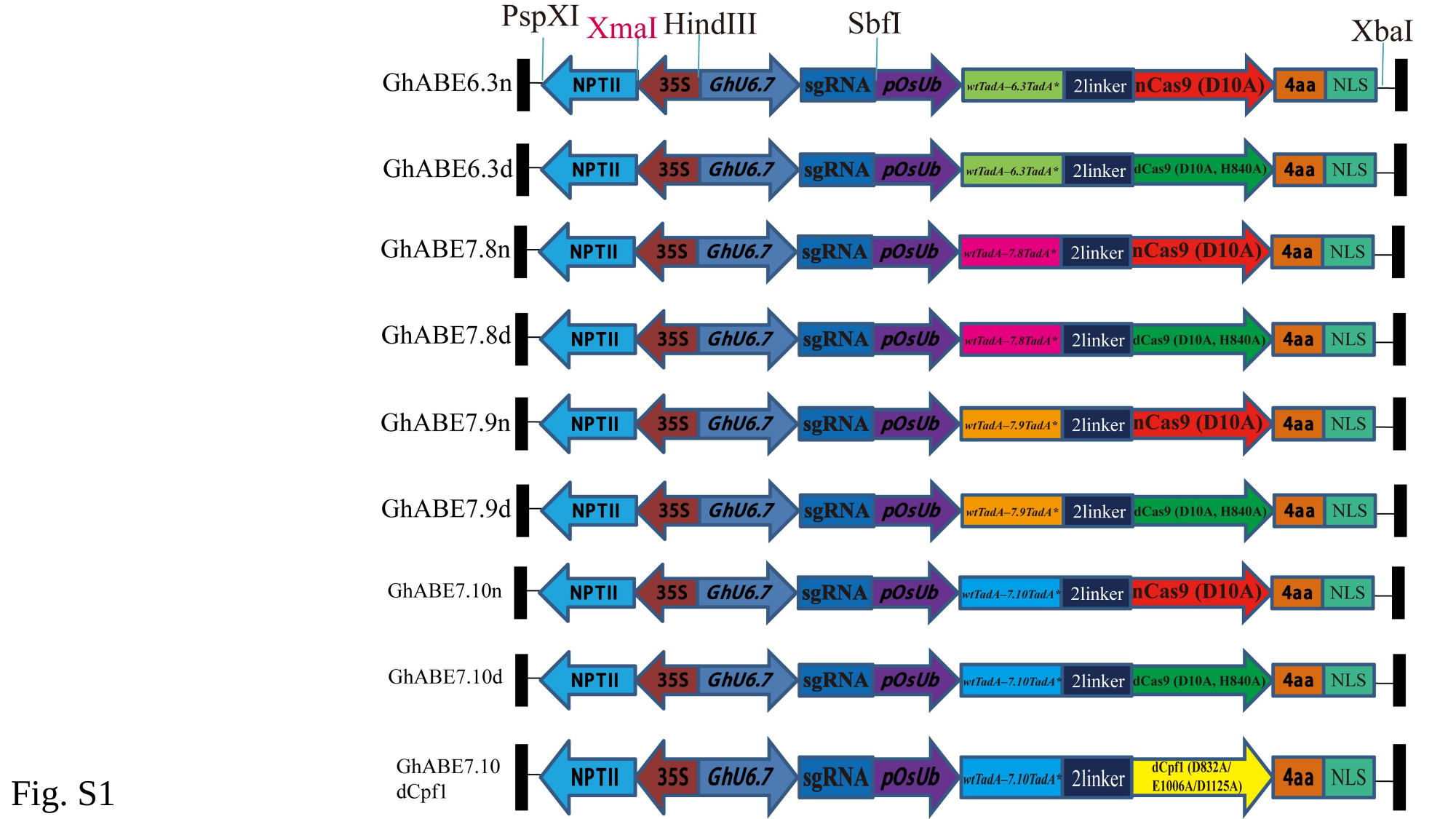

Fig. S1

## Slide 2
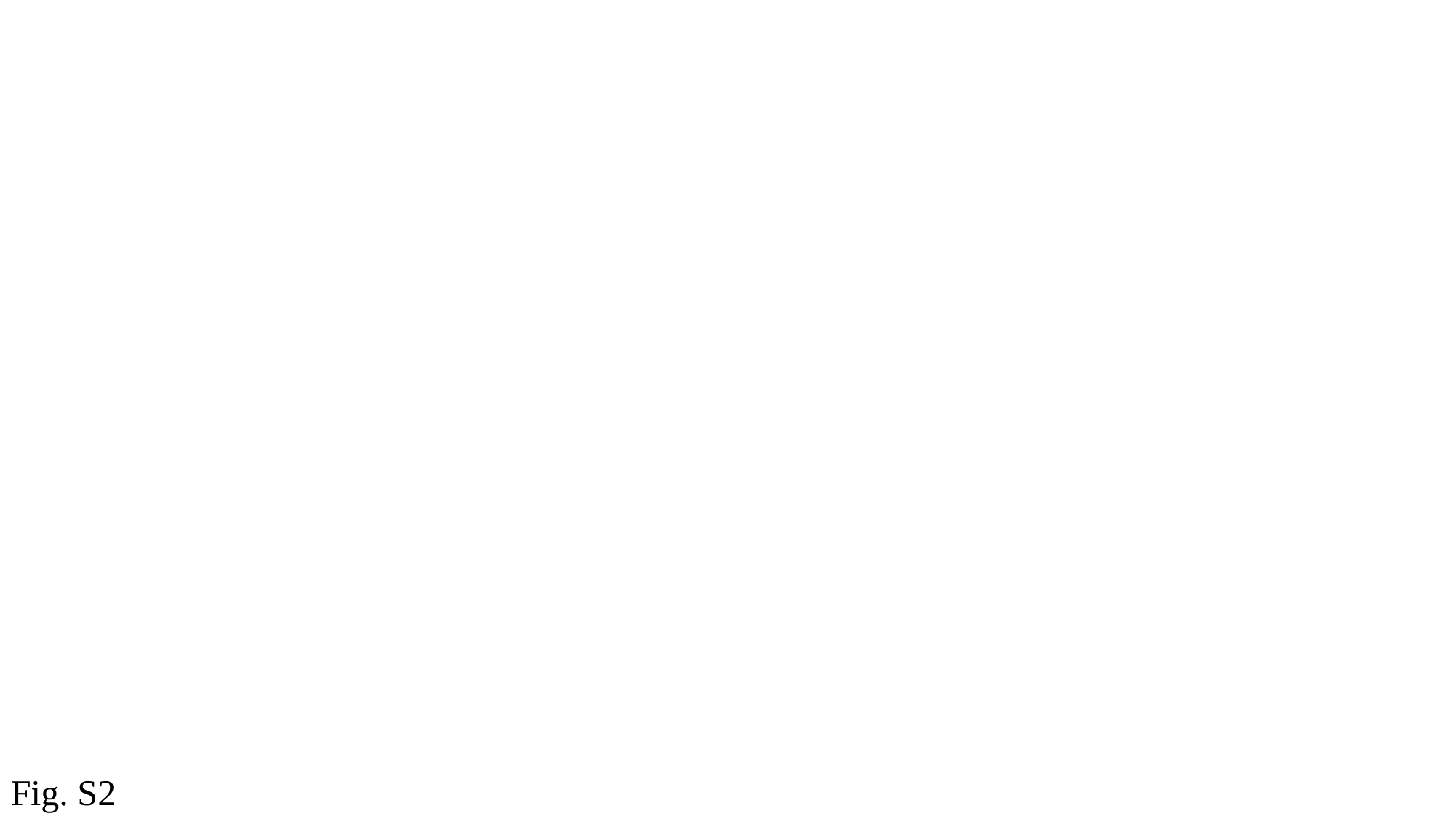

Fig. S2

## Slide 3
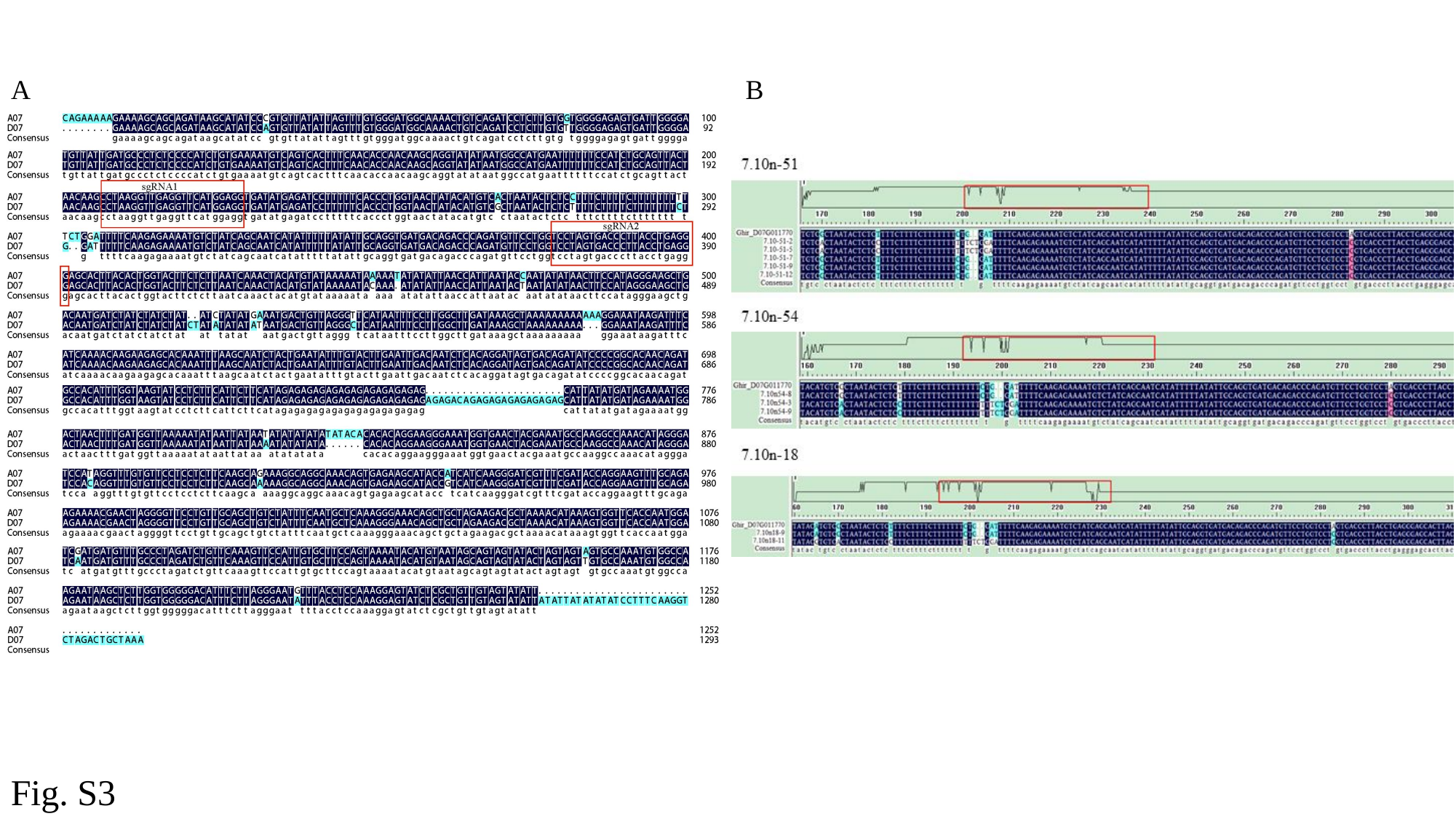

A
B
Fig. S3

## Slide 4
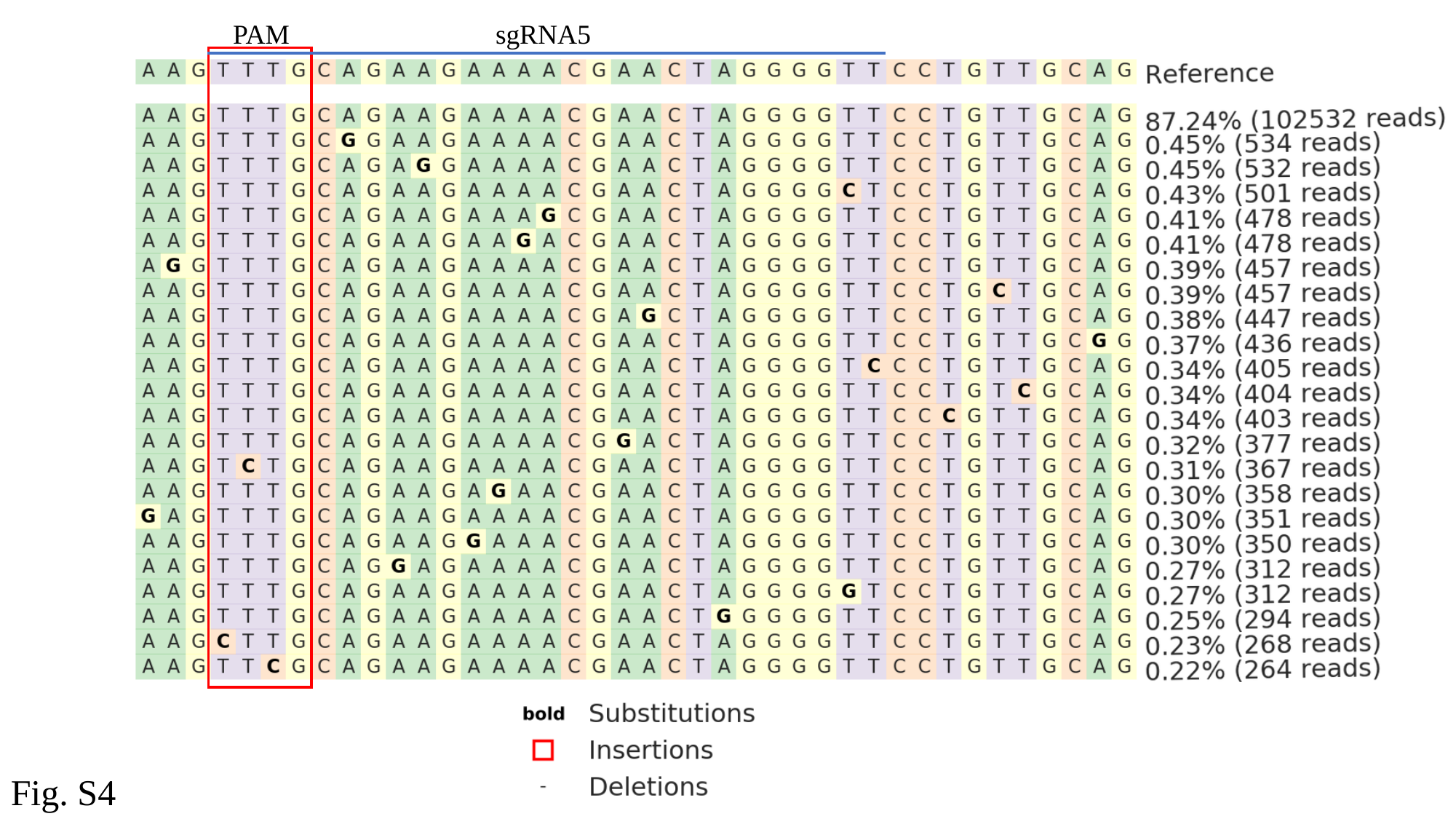

PAM
sgRNA5
Fig. S4

## Slide 5
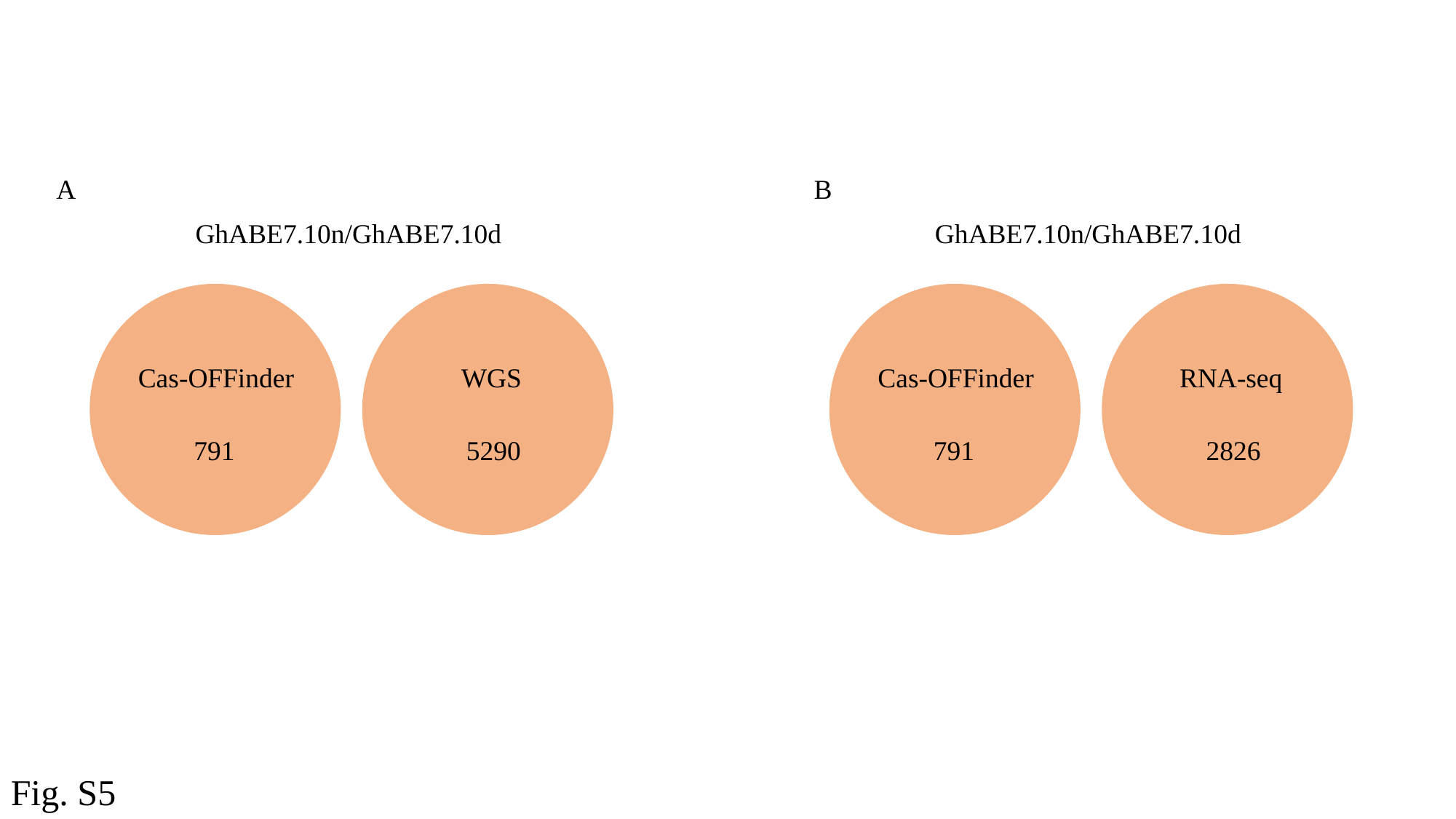

A
B
GhABE7.10n/GhABE7.10d
Cas-OFFinder
WGS
791
5290
GhABE7.10n/GhABE7.10d
Cas-OFFinder
RNA-seq
791
2826
Fig. S5

## Slide 6
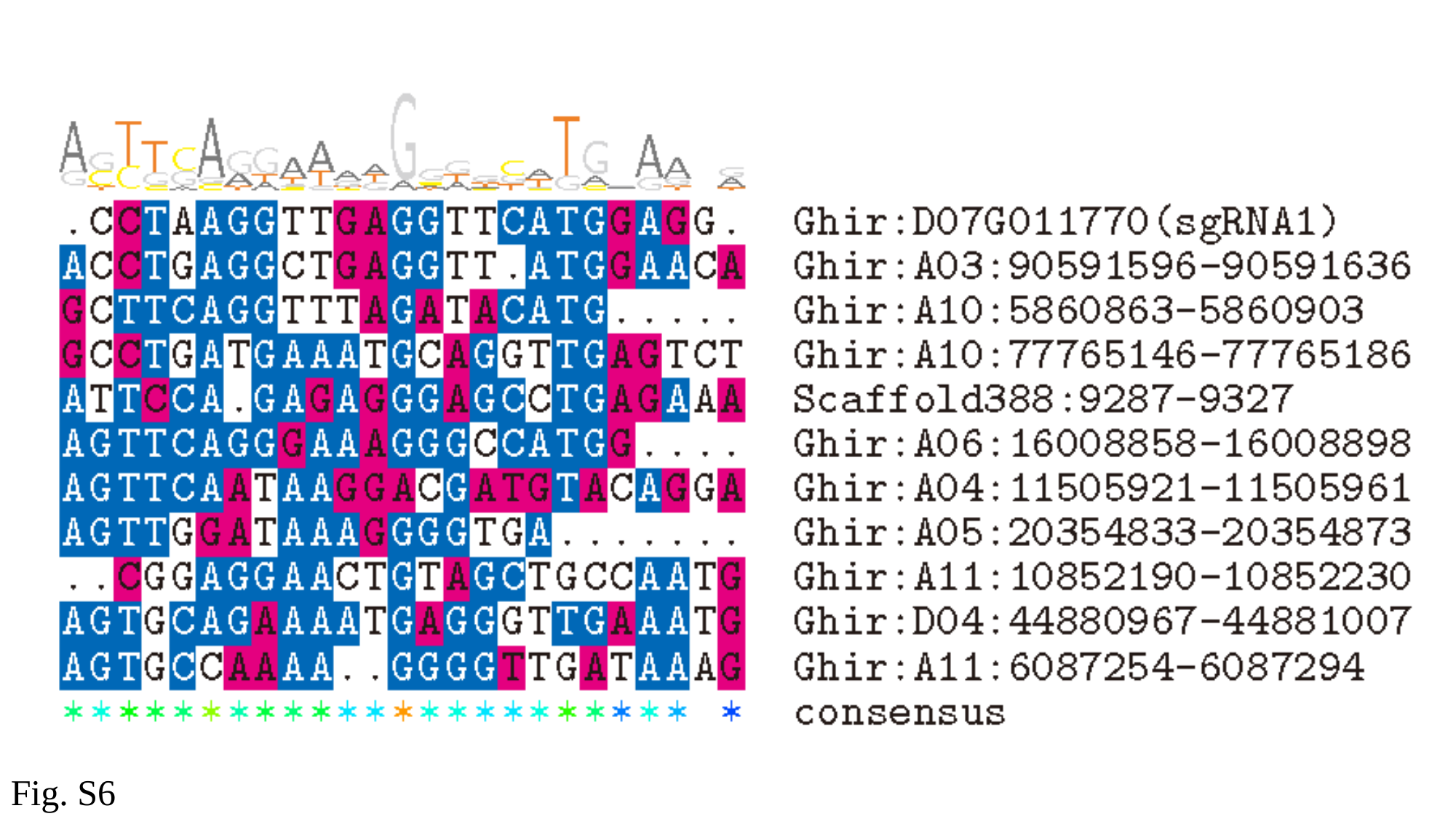

Fig. S6

## Slide 7
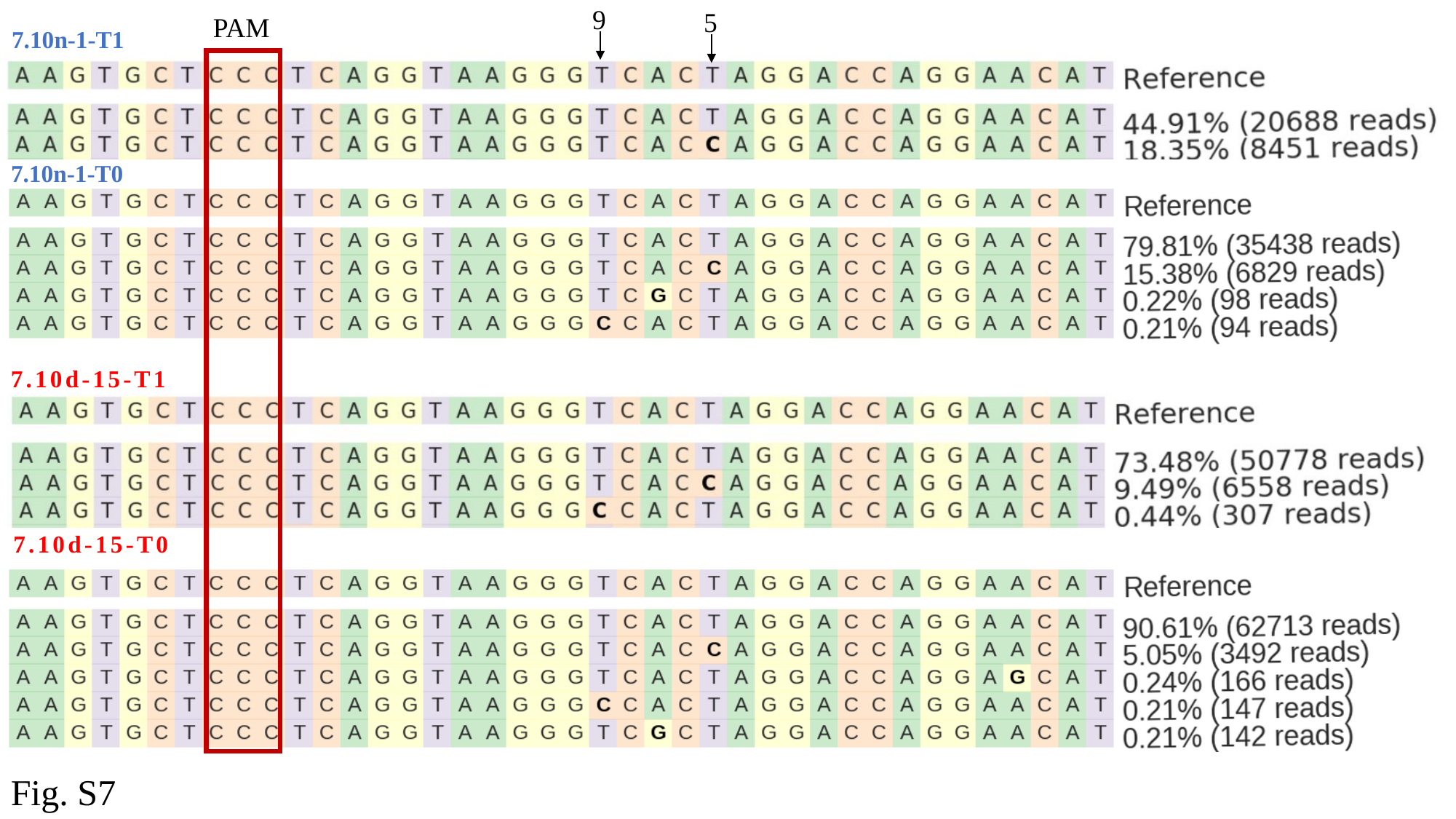

5
9
PAM
# 7.10n-1-T1
7.10n-1-T0
7.10d-15-T1
7.10d-15-T0
Fig. S7

## Slide 8
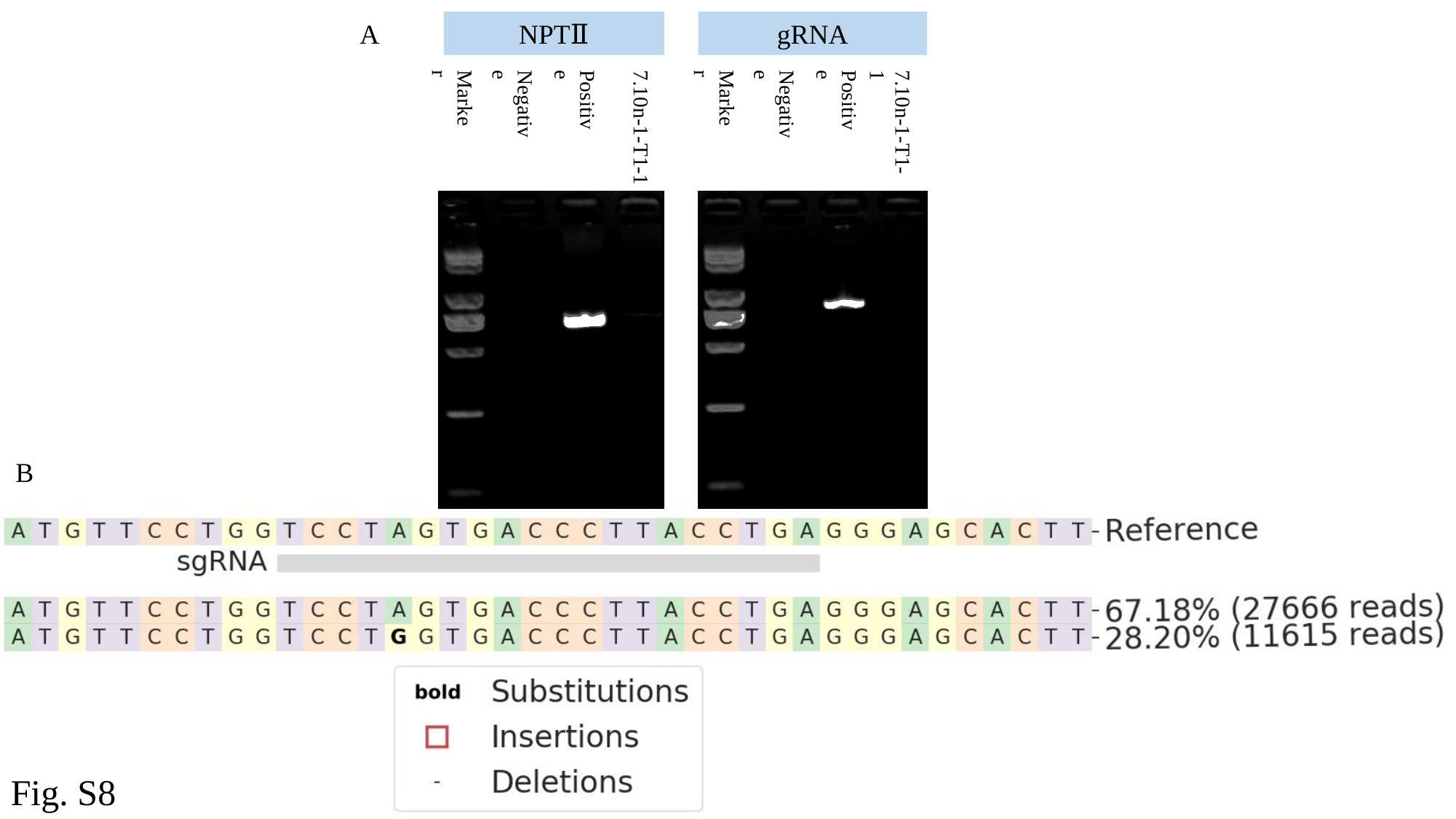

A
NPTⅡ
gRNA
7.10n-1-T1-1
7.10n-1-T1-1
Marker
Negative
Positive
Marker
Negative
Positive
B
Fig. S8

## Slide 9
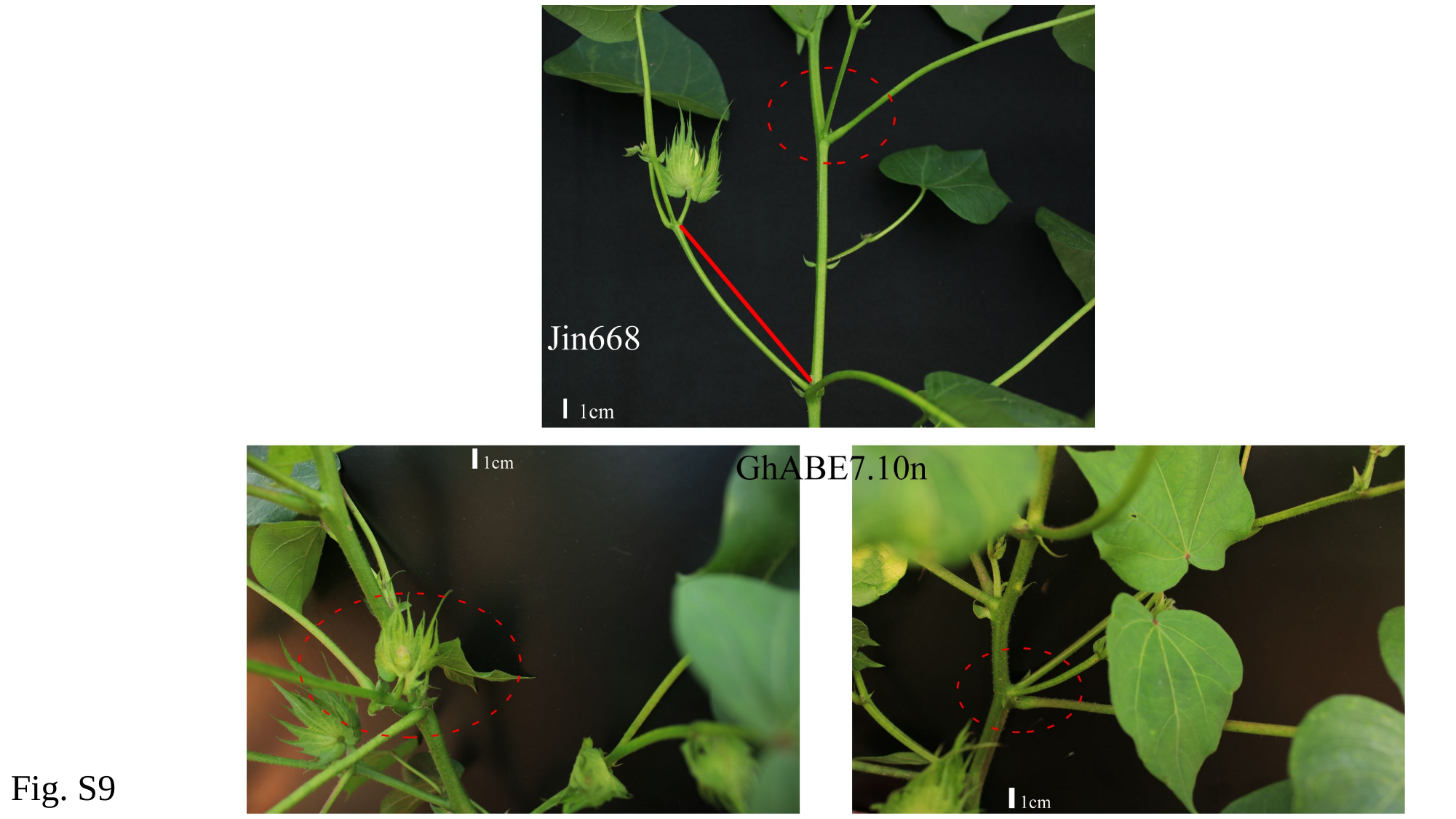

Fig. S9

## Slide 10
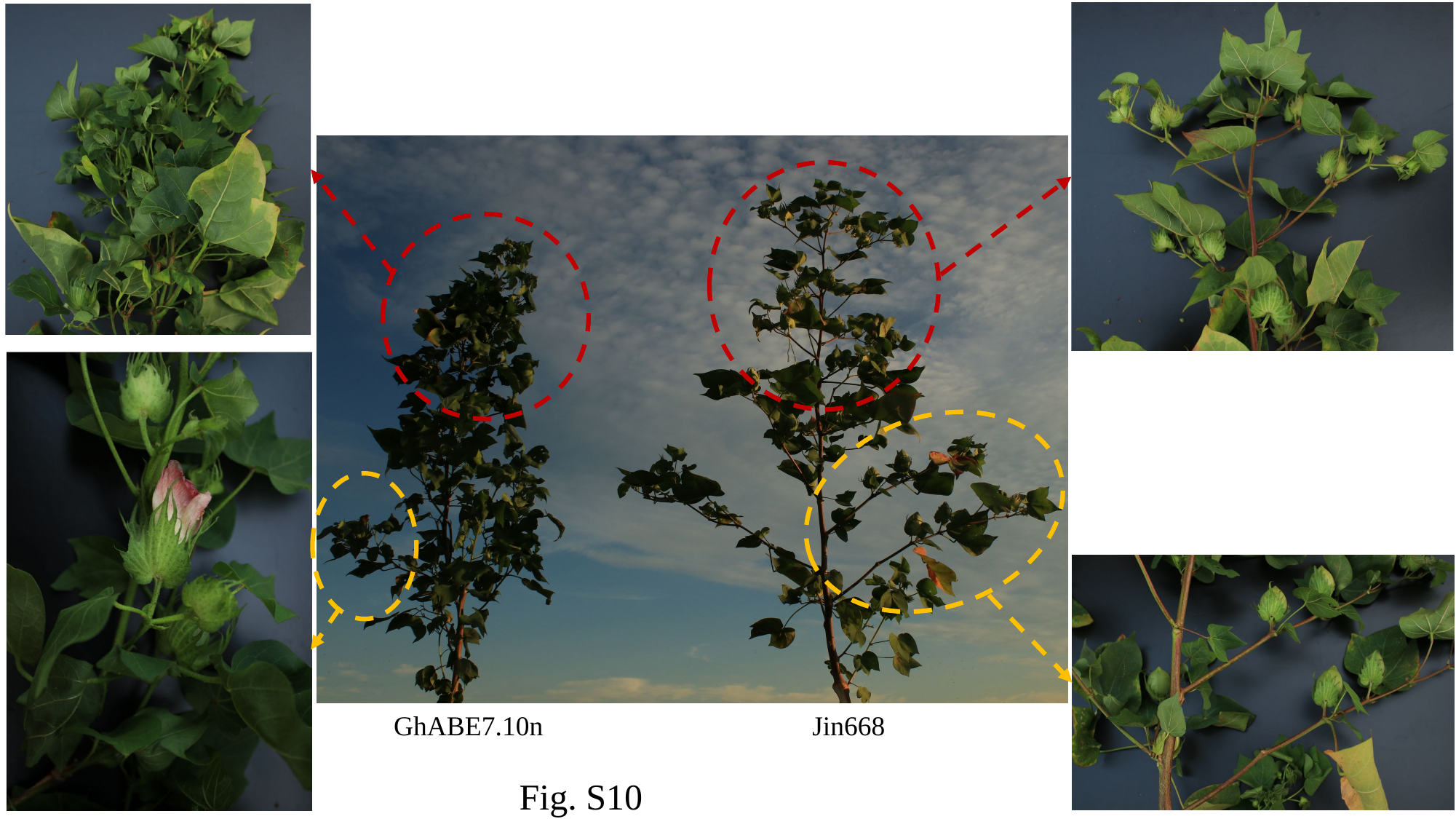

GhABE7.10n Jin668
Fig. S10
