# Supplementary material for: Development of an efficient and precise adenine base editor (ABE) with expanded target range in allotetraploid cotton (Gossypium hirsutum)
Source: BMC Biol. 2022 Feb 15;20:45. doi: 10.1186/s12915-022-01232-3 (PMC8845244; doi:10.1186/s12915-022-01232-3)
Supplement: Supplementary file 3 — Additional file 3:. Supplementary Appendix 1: Sequences of each component of nine GhABEs system. [file 12915_2022_1232_MOESM3_ESM.docx]

# Supplementary FIGURES

**Supplemental Fig. S1**. **Schematic representation of the base editors.**

**Supplemental Fig. S2**. **The identiﬁcation of on-target mutations at GhCLA target sites by targeted deep sequencing.** Base-editing efﬁciency of all A to G conversion within sgRNA3 and sgRNA4 target region using four GhABEs constructs revealed by deep sequencing for single plant.

**Supplemental Fig. S3**. **The different of** **editing efficiency between At and Dt subgenomes of *GhPEBP* gene.** (A) Multiple sequence alignment of sgRNA target regions for *GhPEBE* homologous genes, which shown the SNPs and InDels between At and Dt subgenomes. (B) Sanger sequencing of sgRNA2 in three lines.

**Supplemental Fig. S4**. **Allele compositions following treatment with GhABE7.10dCpf1 at the sgRNA5 of *GhPEBP*.**

**Supplemental Fig. S5**. **Venn diagram analysis of the SNVs identified in ABE base editor together with the off-target sites predicted by Cas-OFFinder.** (A) DNA SNVs from WGS data. (B) RNA SNVs from RNA-seq data.

**Supplemental Fig. S6**. **Similarity between adjacent sequences of off-target RNA SNVs with sgRNA1 target sequences.** The most similarity ten off-target SNVs (top 10) were shown.

**Supplemental Fig. S7**. **The allele composition of T0 and T1 generation at sgRNA2 of *GhPEBP* was treated with GhABE7.10n or GhABE7.10d.**

**Supplemental Fig. S8**. **The Illumina sequencing of transgene-free line isolated from T0 plants.** (A) PCR detection of transgene-free plants. (B) The editing efficiency of the transgene-free plants detected by target deep sequencing.

**Supplemental Fig. S9**. **Comparison of the number of lateral branches and length of fruit nodes of a base-edited T1 plant generated via GhABE7.10n with wild-type Jin668 plant.** Scale bar, 1 cm.

**Supplemental Fig. S10**. **The long-branching WT phenotype (right) and the *GhPEBP* phenotype (left) in upland cotton.** Local area and local magnification are represented by dashed lines of different colors.

# Supplementary TABLE

**Supplemental Table S1.** Primers used for vectors construction.

**Supplemental Table S2.** Primers used for positive test.

**Supplemental Table S3.** Barcode primers for detecting off-target in base editing T0 transgenic plants with deep sequencing for GhPEBE gene.

**Supplemental Table S4.** Barcode primers for detecting off-target in base editing T0 transgenic plants with deep sequencing for GhCLA gene.

**Supplemental Table S5.** Summary of genome-wide potential off-targets predictions by Cas-OFFinder tools for target sgRNA1.

**Supplemental Table S6.** Summary of genome-wide potential off-targets predictions by Cas-OFFinder tools for target sgRNA2.

**Supplemental Table S7.** Expression levels (TPM) of TadA, nCas9 and dCas9 in GhABE7.10 edited plant.

# Appendix

## **Appendix S1: Sequences of each component of nine GhABEs system.**

green = SV40NLS

red = linker(32aa)

black + red = ecTadA (wt)+linker(32aa)+evolved ecTadA*+ linker(32aa)

**brown = nCas9(D10A)**

blue = dCas9 (D10A + H840A)

purple =dCpf1

### GhABE6.3nCas9

(NLS-TadA(wt)–linker(32 aa)–TadA*(6.3)–linker(32 aa)–nCas9–NLS

**ATG**ccaaagaagaagaggaaggtt**TCTGAAGTTGAGTTCTCACATGAATATTGGATGAGACATGCTCTTACATTGGCTAAGAGAGCTTGGGATGAAAGGGAGGTTCCTGTTGGTGCTGTTCTTGTTCATAATAACAGGGTTATTGGTGAAGGATGGAATAGACCTATCGGAAGGCATGATCCAACCGCTCATGCTGAGATTATGGCTCTTAGACAAGGTGGATTGGTTATGCAAAACTATAGGTTGATCGATGCTACACTTTACGTTACCTTGGAGCCTTGTGTTATGTGCGCTGGTGCTATGATTCATTCTAGAATCGGTAGGGTTGTTTTTGGAGCTAGAGATGCTAAGACCGGTGCTGCTGGATCACTTATGGATGTTTTGCATCATCCTGGTATGAATCATAGAGTTGAAATTACTGAGGGAATCCTTGCTGATGAATGTGCTGCTCTTTTGTCTGATTTCTTTAGGATGAGAAGGCAAGAGATTAAAGCTCAAAAGAAAGCTCAATCTTCAACAGATTCTGGTGGATCTTCAGGTGGATCTTCAGGTTCAGAAACTCCTGGAACATCTGAATCAGCTACCCCAGAGTCATCAGGTGGATCATCAGGTGGATCTTCAGAAGTTGAGTTCTCTCATGAGTACTGGATGAGGCACGCTCTTACTCTTGCCAAAAGGGCCTGGGACGAGAGAGAAGTGCCAGTGGGTGCTGTTCTTGTTTTGAATAACAGAGTTATTGGAGAGGGATGGAATAGGTCAATCGGACTTCATGATCCAACAGCCCACGCCGAGATTATGGCCTTGAGGCAAGGTGGATTGGTTATGCAGAACTATAGGCTTATCGATGCTACTCTTTATGTTACATTTGAACCATGCGTGATGTGTGCCGGAGCCATGATCCACTCAAGGATTGGAAGAGTTGTTTTTGGAGTTAGGAATGCTAAAACTGGAGCCGCCGGTTCTCTTATGGATGTGTTGCATTATCCTGGTATGAACCATAGAGTTGAAATTACAGAGGGAATCTTGGCCGACGAGTGCGCTGCTCTTTTGTGCTACTTCTTTAGAATGAGAAGGCAGGTTTTTAACGCCCAAAAGAAAGCCCAATCTTCAACCGACTCTGGTGGATCATCAGGTGGAAGTTCAGGTTCAGAGACCCCTGGAACTTCTGAATCAGCTACTCCAGAGTCCTCAGGTGGATCATCAGGTGGATCGgacaagaagtactcgatcggcctcgccatcgggacgaactcagttggctgggccgtgatcaccgacgagtacaaggtgccctctaagaagttcaaggtcctggggaacaccgaccgccattccatcaagaagaacctcatcggcgctctcctgttcgacagcggggagaccgctgaggctacgaggctcaagagaaccgctaggcgccggtacacgagaaggaagaacaggatctgctacctccaagagattttctccaacgagatggccaaggttgacgattcattcttccaccgcctggaggagtctttcctcgtggaggaggataagaagcacgagcggcatcccatcttcggcaacatcgtggacgaggttgcctaccacgagaagtaccctacgatctaccatctgcggaagaagctcgtggactccaccgataaggcggacctcagactgatctacctcgctctggcccacatgatcaagttccgcggccatttcctgatcgagggggatctcaacccagacaacagcgatgttgacaagctgttcatccaactcgtgcagacctacaaccaactcttcgaggagaacccgatcaacgcctctggcgtggacgcgaaggctatcctgtccgcgaggctctcgaagtccaggaggctggagaacctgatcgctcagctcccaggcgagaagaagaacggcctgttcgggaacctcatcgctctcagcctggggctcaccccgaacttcaagtcgaacttcgatctcgctgaggacgccaagctgcaactctccaaggacacctacgacgatgacctcgataacctcctggcccagatcggcgatcaatacgcggacctgttcctcgctgccaagaacctgtcggacgccatcctcctgtcagatatcctccgcgtgaacaccgagatcacgaaggctccactctctgcctccatgatcaagcgctacgacgagcaccatcaggatctgaccctcctgaaggcgctggtccgccaacagctcccggagaagtacaaggagattttcttcgatcagtcgaagaacggctacgctgggtacatcgacggcggggcctcacaagaggagttctacaagttcatcaagccaatcctggagaagatggacggcacggaggagctcctggtgaagctcaacagggaggacctcctgcggaagcagagaaccttcgataacggcagcatcccccaccaaatccatctcggggagctgcacgccatcctgagaaggcaagaggacttctaccctttcctcaaggataaccgggagaagatcgagaagatcctgaccttcagaatcccatactacgtcggccctctcgcgcgggggaactcaagattcgcttggatgacccgcaagtctgaggagaccatcacgccgtggaacttcgaggaggtggtggacaagggcgctagcgctcagtcgttcatcgagaggatgaccaacttcgacaagaacctgcccaacgagaaggtgctccctaagcactcgctcctgtacgagtacttcaccgtctacaacgagctcacgaaggtgaagtacgtcaccgagggcatgcgcaagccagcgttcctgtccggggagcagaagaaggctatcgtggacctcctgttcaagaccaaccggaaggtcacggttaagcaactcaaggaggactacttcaagaagatcgagtgcttcgattcggtcgagatcagcggcgttgaggaccgcttcaacgccagcctcgggacctaccacgatctcctgaagatcatcaaggataaggacttcctggacaacgaggagaacgaggatatcctggaggacatcgtgctgaccctcacgctgttcgaggacagggagatgatcgaggagcgcctgaagacgtacgcccatctcttcgatgacaaggtcatgaagcaactcaagcgccggagatacaccggctgggggaggctgtcccgcaagctcatcaacggcatccgggacaagcagtccgggaagaccatcctcgacttcctcaagagcgatggcttcgccaacaggaacttcatgcaactgatccacgatgacagcctcaccttcaaggaggatatccaaaaggctcaagtgagcggccagggggactcgctgcacgagcatatcgcgaacctcgctggctcccccgcgatcaagaagggcatcctccagaccgtgaaggttgtggacgagctcgtgaaggtcatgggccggcacaagcctgagaacatcgtcatcgagatggccagagagaaccaaaccacgcagaaggggcaaaagaactctagggagcgcatgaagcgcatcgaggagggcatcaaggagctggggtcccaaatcctcaaggagcacccagtggagaacacccaactgcagaacgagaagctctacctgtactacctccagaacggcagggatatgtacgtggaccaagagctggatatcaaccgcctcagcgattacgacgtcgatcatatcgttccccagtctttcctgaaggatgactccatcgacaacaaggtcctcaccaggtcggacaagaaccgcggcaagtcagataacgttccatctgaggaggtcgttaagaagatgaagaactactggaggcagctcctgaacgccaagctgatcacgcaaaggaagttcgacaacctcaccaaggctgagagaggcgggctctcagagctggacaaggccggcttcatcaagcggcagctggtcgagaccagacaaatcacgaagcacgttgcgcaaatcctcgactctcggatgaacacgaagtacgatgagaacgacaagctgatcagggaggttaaggtgatcaccctgaagtctaagctcgtctccgacttcaggaaggatttccagttctacaaggttcgcgagatcaacaactaccaccatgcccatgacgcttacctcaacgctgtggtcggcaccgctctgatcaagaagtacccaaagctggagtccgagttcgtgtacggggactacaaggtttacgatgtgcgcaagatgatcgccaagtcggagcaagagatcggcaaggctaccgccaagtacttcttctactcaaacatcatgaacttcttcaagaccgagatcacgctggccaacggcgagatccggaagagaccgctcatcgagaccaacggcgagacgggggagatcgtgtgggacaagggcagggatttcgcgaccgtccgcaaggttctctccatgccccaggtgaacatcgtcaagaagaccgaggtccaaacgggcgggttctcaaaggagtctatcctgcctaagcggaacagcgacaagctcatcgccagaaagaaggactgggacccaaagaagtacggcgggttcgacagccctaccgtggcctactcggtcctggttgtggcgaaggttgagaagggcaagtccaagaagctcaagagcgtgaaggagctcctggggatcaccatcatggagaggtccagcttcgagaagaacccaatcgacttcctggaggccaagggctacaaggaggtgaagaaggacctgatcatcaagctcccgaagtactctctcttcgagctggagaacggcaggaagagaatgctggcttccgctggcgagctccagaaggggaacgagctcgcgctgccaagcaagtacgtgaacttcctctacctggcttcccactacgagaagctcaagggcagcccggaggacaacgagcaaaagcagctgttcgtcgagcagcacaagcattacctcgacgagatcatcgagcaaatctccgagttcagcaagcgcgtgatcctcgccgacgcgaacctggataaggtcctctccgcctacaacaagcaccgggacaagcccatcagagagcaagcggagaacatcatccatctcttcaccctgacgaacctcggcgctcctgctgctttcaagtacttcgacaccacgatcgatcggaagagatacacctccacgaaggaggtcctggacgcgaccctcatccaccagtcgatcaccggcctgtacgagacgaggatcgacctctcacaactcggcggggataagagacccgcagcaaccaagaaggcagggcaagcaaagaagaagaagacgcgt**ccaaagaagaagcggaaggtg

### GhABE7.8nCas9

(NLS-TadA(wt)–linker(32 aa)–TadA*(7.8)–linker(32 aa)–nCas9–NLS

**ATG**ccaaagaagaagaggaaggtt**TCTGAAGTTGAGTTCTCACATGAATATTGGATGAGACATGCTCTTACATTGGCTAAGAGAGCTTGGGATGAAAGGGAGGTTCCTGTTGGTGCTGTTCTTGTTCATAATAACAGGGTTATTGGTGAAGGATGGAACAGACCTATCGGAAGGCATGATCCAACCGCTCATGCTGAGATTATGGCTCTTAGACAAGGTGGATTGGTTATGCAAAATTATAGGTTGATCGATGCTACACTTTACGTTACCTTGGAGCCTTGTGTTATGTGCGCTGGTGCTATGATTCATTCTAGAATCGGTAGGGTTGTTTTTGGAGCTAGAGATGCTAAGACCGGTGCTGCTGGATCACTTATGGATGTTTTGCATCATCCTGGTATGAACCATAGAGTTGAAATTACTGAGGGAATCCTTGCTGATGAATGTGCTGCTCTTTTGTCTGATTTCTTTAGGATGAGAAGGCAAGAGATTAAAGCTCAAAAGAAAGCTCAATCTTCAACAGAT**TCTGGTGGATCTTCAGGTGGATCTTCAGGTTCAGAAACTCCTGGAACATCTGAATCAGCTACCCCAGAGTCATCAGGTGGATCATCAGGTGGATCT**TCAGAGGTGGAGTTCTCACATGAGTACTGGATGAGGCACGCTCTTACTTTGGCTAAGAGAGCTCTTGACGAGAGAGAAGTGCCAGTGGGAGCTGTTCTTGTTTTGAATAACAGAGTTATTGGAGAGGGATGGAACAGGGCTATCGGATTGCATGATCCAACAGCCCACGCCGAGATTATGGCCTTGAGGCAAGGTGGATTGGTTATGCAGAATTATAGGCTTATCGATGCTACTCTTTATGTTACATTTGAACCATGCGTGATGTGTGCCGGAGCCATGATCCACTCAAGGATTGGAAGAGTTGTTTTTGGAGTTAGGAATGCTAAAACTGGAGCCGCCGGTTCTCTTATGGATGTGTTGCATTATCCTGGTATGAATCATAGAGTTGAAATTACAGAGGGAATCTTGGCTGATGAATGTAACGCTCTTTTGTGCTACTTCTTTAGAATGAGAAGGCAGGTTTTTAATGCCCAAAAGAAAGCCCAATCTTCAACCGAC**TCTGGTGGATCATCAGGTGGAAGTTCAGGTTCAGAGACCCCTGGAACTTCTGAATCAGCTACTCCAGAGTCCTCAGGTGGATCATCAGGTGGATCG**gacaagaagtactcgatcggcctcgccatcgggacgaactcagttggctgggccgtgatcaccgacgagtacaaggtgccctctaagaagttcaaggtcctggggaacaccgaccgccattccatcaagaagaacctcatcggcgctctcctgttcgacagcggggagaccgctgaggctacgaggctcaagagaaccgctaggcgccggtacacgagaaggaagaacaggatctgctacctccaagagattttctccaacgagatggccaaggttgacgattcattcttccaccgcctggaggagtctttcctcgtggaggaggataagaagcacgagcggcatcccatcttcggcaacatcgtggacgaggttgcctaccacgagaagtaccctacgatctaccatctgcggaagaagctcgtggactccaccgataaggcggacctcagactgatctacctcgctctggcccacatgatcaagttccgcggccatttcctgatcgagggggatctcaacccagacaacagcgatgttgacaagctgttcatccaactcgtgcagacctacaaccaactcttcgaggagaacccgatcaacgcctctggcgtggacgcgaaggctatcctgtccgcgaggctctcgaagtccaggaggctggagaacctgatcgctcagctcccaggcgagaagaagaacggcctgttcgggaacctcatcgctctcagcctggggctcaccccgaacttcaagtcgaacttcgatctcgctgaggacgccaagctgcaactctccaaggacacctacgacgatgacctcgataacctcctggcccagatcggcgatcaatacgcggacctgttcctcgctgccaagaacctgtcggacgccatcctcctgtcagatatcctccgcgtgaacaccgagatcacgaaggctccactctctgcctccatgatcaagcgctacgacgagcaccatcaggatctgaccctcctgaaggcgctggtccgccaacagctcccggagaagtacaaggagattttcttcgatcagtcgaagaacggctacgctgggtacatcgacggcggggcctcacaagaggagttctacaagttcatcaagccaatcctggagaagatggacggcacggaggagctcctggtgaagctcaacagggaggacctcctgcggaagcagagaaccttcgataacggcagcatcccccaccaaatccatctcggggagctgcacgccatcctgagaaggcaagaggacttctaccctttcctcaaggataaccgggagaagatcgagaagatcctgaccttcagaatcccatactacgtcggccctctcgcgcgggggaactcaagattcgcttggatgacccgcaagtctgaggagaccatcacgccgtggaacttcgaggaggtggtggacaagggcgctagcgctcagtcgttcatcgagaggatgaccaacttcgacaagaacctgcccaacgagaaggtgctccctaagcactcgctcctgtacgagtacttcaccgtctacaacgagctcacgaaggtgaagtacgtcaccgagggcatgcgcaagccagcgttcctgtccggggagcagaagaaggctatcgtggacctcctgttcaagaccaaccggaaggtcacggttaagcaactcaaggaggactacttcaagaagatcgagtgcttcgattcggtcgagatcagcggcgttgaggaccgcttcaacgccagcctcgggacctaccacgatctcctgaagatcatcaaggataaggacttcctggacaacgaggagaacgaggatatcctggaggacatcgtgctgaccctcacgctgttcgaggacagggagatgatcgaggagcgcctgaagacgtacgcccatctcttcgatgacaaggtcatgaagcaactcaagcgccggagatacaccggctgggggaggctgtcccgcaagctcatcaacggcatccgggacaagcagtccgggaagaccatcctcgacttcctcaagagcgatggcttcgccaacaggaacttcatgcaactgatccacgatgacagcctcaccttcaaggaggatatccaaaaggctcaagtgagcggccagggggactcgctgcacgagcatatcgcgaacctcgctggctcccccgcgatcaagaagggcatcctccagaccgtgaaggttgtggacgagctcgtgaaggtcatgggccggcacaagcctgagaacatcgtcatcgagatggccagagagaaccaaaccacgcagaaggggcaaaagaactctagggagcgcatgaagcgcatcgaggagggcatcaaggagctggggtcccaaatcctcaaggagcacccagtggagaacacccaactgcagaacgagaagctctacctgtactacctccagaacggcagggatatgtacgtggaccaagagctggatatcaaccgcctcagcgattacgacgtcgatcatatcgttccccagtctttcctgaaggatgactccatcgacaacaaggtcctcaccaggtcggacaagaaccgcggcaagtcagataacgttccatctgaggaggtcgttaagaagatgaagaactactggaggcagctcctgaacgccaagctgatcacgcaaaggaagttcgacaacctcaccaaggctgagagaggcgggctctcagagctggacaaggccggcttcatcaagcggcagctggtcgagaccagacaaatcacgaagcacgttgcgcaaatcctcgactctcggatgaacacgaagtacgatgagaacgacaagctgatcagggaggttaaggtgatcaccctgaagtctaagctcgtctccgacttcaggaaggatttccagttctacaaggttcgcgagatcaacaactaccaccatgcccatgacgcttacctcaacgctgtggtcggcaccgctctgatcaagaagtacccaaagctggagtccgagttcgtgtacggggactacaaggtttacgatgtgcgcaagatgatcgccaagtcggagcaagagatcggcaaggctaccgccaagtacttcttctactcaaacatcatgaacttcttcaagaccgagatcacgctggccaacggcgagatccggaagagaccgctcatcgagaccaacggcgagacgggggagatcgtgtgggacaagggcagggatttcgcgaccgtccgcaaggttctctccatgccccaggtgaacatcgtcaagaagaccgaggtccaaacgggcgggttctcaaaggagtctatcctgcctaagcggaacagcgacaagctcatcgccagaaagaaggactgggacccaaagaagtacggcgggttcgacagccctaccgtggcctactcggtcctggttgtggcgaaggttgagaagggcaagtccaagaagctcaagagcgtgaaggagctcctggggatcaccatcatggagaggtccagcttcgagaagaacccaatcgacttcctggaggccaagggctacaaggaggtgaagaaggacctgatcatcaagctcccgaagtactctctcttcgagctggagaacggcaggaagagaatgctggcttccgctggcgagctccagaaggggaacgagctcgcgctgccaagcaagtacgtgaacttcctctacctggcttcccactacgagaagctcaagggcagcccggaggacaacgagcaaaagcagctgttcgtcgagcagcacaagcattacctcgacgagatcatcgagcaaatctccgagttcagcaagcgcgtgatcctcgccgacgcgaacctggataaggtcctctccgcctacaacaagcaccgggacaagcccatcagagagcaagcggagaacatcatccatctcttcaccctgacgaacctcggcgctcctgctgctttcaagtacttcgacaccacgatcgatcggaagagatacacctccacgaaggaggtcctggacgcgaccctcatccaccagtcgatcaccggcctgtacgagacgaggatcgacctctcacaactcggcggggataagagacccgcagcaaccaagaaggcagggcaagcaaagaagaagaagacgcgt**ccaaagaagaagcggaaggtg

### GhABE7.9nCas9

(NLS-TadA(wt)–linker(32 aa)–TadA*(7.9)–linker(32 aa)–nCas9–NLS

**ATG**ccaaagaagaagaggaaggtt**TCTGAAGTTGAGTTCTCACATGAATATTGGATGAGACATGCTCTTACATTGGCTAAGAGAGCTTGGGATGAAAGGGAGGTTCCTGTTGGTGCTGTTCTTGTTCATAATAACAGGGTTATTGGTGAAGGATGGAACAGACCTATCGGAAGGCATGATCCAACCGCTCATGCTGAGATTATGGCTCTTAGACAAGGTGGATTGGTTATGCAAAATTATAGGTTGATCGATGCTACACTTTACGTTACCTTGGAGCCTTGTGTTATGTGCGCTGGTGCTATGATTCATTCTAGAATCGGTAGGGTTGTTTTTGGAGCTAGAGATGCTAAGACCGGTGCTGCTGGATCACTTATGGATGTTTTGCATCATCCTGGTATGAACCATAGAGTTGAAATTACTGAGGGAATCCTTGCTGATGAATGTGCTGCTCTTTTGTCTGATTTCTTTAGGATGAGAAGGCAAGAGATTAAAGCTCAAAAGAAAGCTCAATCTTCAACAGAT**TCTGGTGGATCTTCAGGTGGATCTTCAGGTTCAGAAACTCCTGGAACATCTGAATCAGCTACCCCAGAGTCATCAGGTGGATCATCAGGTGGATCT**TCAGAGGTGGAGTTCTCACATGAGTACTGGATGAGGCATGCTCTTACTTTGGCTAAGAGAGCTCTTGACGAGAGAGAAGTGCCAGTGGGAGCTGTTCTTGTTTTGAATAACAGAGTTATTGGAGAGGGATGGAACAGGGCTATCGGATTGCATGATCCAACAGCCCACGCCGAGATTATGGCCTTGAGGCAAGGTGGATTGGTTATGCAGAATTATAGGCTTATCGATGCTACTCTTTATGTTACATTTGAACCATGCGTGATGTGTGCCGGAGCCATGATCCACTCAAGGATTGGAAGGGTTGTTTTTGGAGTTAGAAATGCTAAAACTGGAGCCGCCGGTTCTCTTATGGATGTGTTGCATTATCCTGGTATGAATCATAGGGTTGAAATTACAGAGGGAATCTTGGCTGATGAATGTAACGCTCTTTTGTGCTACTTCTTTAGAATGCCAAGACAAGTTTTTAATGCCCAAAAGAAAGCCCAATCTTCAACCGAC**TCTGGTGGATCATCAGGTGGAAGTTCAGGTTCAGAGACCCCTGGAACTTCTGAATCAGCTACTCCAGAGTCCTCAGGTGGATCATCAGGTGGATCG**gacaagaagtactcgatcggcctcgccatcgggacgaactcagttggctgggccgtgatcaccgacgagtacaaggtgccctctaagaagttcaaggtcctggggaacaccgaccgccattccatcaagaagaacctcatcggcgctctcctgttcgacagcggggagaccgctgaggctacgaggctcaagagaaccgctaggcgccggtacacgagaaggaagaacaggatctgctacctccaagagattttctccaacgagatggccaaggttgacgattcattcttccaccgcctggaggagtctttcctcgtggaggaggataagaagcacgagcggcatcccatcttcggcaacatcgtggacgaggttgcctaccacgagaagtaccctacgatctaccatctgcggaagaagctcgtggactccaccgataaggcggacctcagactgatctacctcgctctggcccacatgatcaagttccgcggccatttcctgatcgagggggatctcaacccagacaacagcgatgttgacaagctgttcatccaactcgtgcagacctacaaccaactcttcgaggagaacccgatcaacgcctctggcgtggacgcgaaggctatcctgtccgcgaggctctcgaagtccaggaggctggagaacctgatcgctcagctcccaggcgagaagaagaacggcctgttcgggaacctcatcgctctcagcctggggctcaccccgaacttcaagtcgaacttcgatctcgctgaggacgccaagctgcaactctccaaggacacctacgacgatgacctcgataacctcctggcccagatcggcgatcaatacgcggacctgttcctcgctgccaagaacctgtcggacgccatcctcctgtcagatatcctccgcgtgaacaccgagatcacgaaggctccactctctgcctccatgatcaagcgctacgacgagcaccatcaggatctgaccctcctgaaggcgctggtccgccaacagctcccggagaagtacaaggagattttcttcgatcagtcgaagaacggctacgctgggtacatcgacggcggggcctcacaagaggagttctacaagttcatcaagccaatcctggagaagatggacggcacggaggagctcctggtgaagctcaacagggaggacctcctgcggaagcagagaaccttcgataacggcagcatcccccaccaaatccatctcggggagctgcacgccatcctgagaaggcaagaggacttctaccctttcctcaaggataaccgggagaagatcgagaagatcctgaccttcagaatcccatactacgtcggccctctcgcgcgggggaactcaagattcgcttggatgacccgcaagtctgaggagaccatcacgccgtggaacttcgaggaggtggtggacaagggcgctagcgctcagtcgttcatcgagaggatgaccaacttcgacaagaacctgcccaacgagaaggtgctccctaagcactcgctcctgtacgagtacttcaccgtctacaacgagctcacgaaggtgaagtacgtcaccgagggcatgcgcaagccagcgttcctgtccggggagcagaagaaggctatcgtggacctcctgttcaagaccaaccggaaggtcacggttaagcaactcaaggaggactacttcaagaagatcgagtgcttcgattcggtcgagatcagcggcgttgaggaccgcttcaacgccagcctcgggacctaccacgatctcctgaagatcatcaaggataaggacttcctggacaacgaggagaacgaggatatcctggaggacatcgtgctgaccctcacgctgttcgaggacagggagatgatcgaggagcgcctgaagacgtacgcccatctcttcgatgacaaggtcatgaagcaactcaagcgccggagatacaccggctgggggaggctgtcccgcaagctcatcaacggcatccgggacaagcagtccgggaagaccatcctcgacttcctcaagagcgatggcttcgccaacaggaacttcatgcaactgatccacgatgacagcctcaccttcaaggaggatatccaaaaggctcaagtgagcggccagggggactcgctgcacgagcatatcgcgaacctcgctggctcccccgcgatcaagaagggcatcctccagaccgtgaaggttgtggacgagctcgtgaaggtcatgggccggcacaagcctgagaacatcgtcatcgagatggccagagagaaccaaaccacgcagaaggggcaaaagaactctagggagcgcatgaagcgcatcgaggagggcatcaaggagctggggtcccaaatcctcaaggagcacccagtggagaacacccaactgcagaacgagaagctctacctgtactacctccagaacggcagggatatgtacgtggaccaagagctggatatcaaccgcctcagcgattacgacgtcgatcatatcgttccccagtctttcctgaaggatgactccatcgacaacaaggtcctcaccaggtcggacaagaaccgcggcaagtcagataacgttccatctgaggaggtcgttaagaagatgaagaactactggaggcagctcctgaacgccaagctgatcacgcaaaggaagttcgacaacctcaccaaggctgagagaggcgggctctcagagctggacaaggccggcttcatcaagcggcagctggtcgagaccagacaaatcacgaagcacgttgcgcaaatcctcgactctcggatgaacacgaagtacgatgagaacgacaagctgatcagggaggttaaggtgatcaccctgaagtctaagctcgtctccgacttcaggaaggatttccagttctacaaggttcgcgagatcaacaactaccaccatgcccatgacgcttacctcaacgctgtggtcggcaccgctctgatcaagaagtacccaaagctggagtccgagttcgtgtacggggactacaaggtttacgatgtgcgcaagatgatcgccaagtcggagcaagagatcggcaaggctaccgccaagtacttcttctactcaaacatcatgaacttcttcaagaccgagatcacgctggccaacggcgagatccggaagagaccgctcatcgagaccaacggcgagacgggggagatcgtgtgggacaagggcagggatttcgcgaccgtccgcaaggttctctccatgccccaggtgaacatcgtcaagaagaccgaggtccaaacgggcgggttctcaaaggagtctatcctgcctaagcggaacagcgacaagctcatcgccagaaagaaggactgggacccaaagaagtacggcgggttcgacagccctaccgtggcctactcggtcctggttgtggcgaaggttgagaagggcaagtccaagaagctcaagagcgtgaaggagctcctggggatcaccatcatggagaggtccagcttcgagaagaacccaatcgacttcctggaggccaagggctacaaggaggtgaagaaggacctgatcatcaagctcccgaagtactctctcttcgagctggagaacggcaggaagagaatgctggcttccgctggcgagctccagaaggggaacgagctcgcgctgccaagcaagtacgtgaacttcctctacctggcttcccactacgagaagctcaagggcagcccggaggacaacgagcaaaagcagctgttcgtcgagcagcacaagcattacctcgacgagatcatcgagcaaatctccgagttcagcaagcgcgtgatcctcgccgacgcgaacctggataaggtcctctccgcctacaacaagcaccgggacaagcccatcagagagcaagcggagaacatcatccatctcttcaccctgacgaacctcggcgctcctgctgctttcaagtacttcgacaccacgatcgatcggaagagatacacctccacgaaggaggtcctggacgcgaccctcatccaccagtcgatcaccggcctgtacgagacgaggatcgacctctcacaactcggcggggataagagacccgcagcaaccaagaaggcagggcaagcaaagaagaagaagacgcgt**ccaaagaagaagcggaaggtg

### GhABE7.10nCas9

(NLS-TadA(wt)–linker(32 aa)–TadA*(7.10)–linker(32 aa)–nCas9–NLS

**ATG**ccaaagaagaagaggaaggtt**TCTGAAGTTGAGTTCTCACATGAATATTGGATGAGACATGCTCTTACATTGGCTAAGAGAGCTTGGGATGAAAGGGAGGTTCCTGTTGGTGCTGTTCTTGTTCATAATAACAGGGTTATTGGTGAAGGATGGAATAGACCTATCGGAAGGCATGATCCAACCGCTCATGCTGAGATTATGGCTCTTAGACAAGGTGGATTGGTTATGCAAAACTATAGGTTGATCGATGCTACACTTTACGTTACCTTGGAGCCTTGTGTTATGTGCGCTGGTGCTATGATTCATTCTAGAATCGGTAGGGTTGTTTTTGGAGCTAGAGATGCTAAGACCGGTGCTGCTGGATCACTTATGGATGTTTTGCATCATCCTGGTATGAATCATAGAGTTGAAATTACTGAGGGAATCCTTGCTGATGAATGTGCTGCTCTTTTGTCTGATTTCTTTAGGATGAGAAGGCAAGAGATTAAAGCTCAAAAGAAAGCTCAATCTTCAACAGAT**TCTGGTGGATCTTCAGGTGGATCTTCAGGTTCAGAAACTCCTGGAACATCTGAATCAGCTACCCCAGAGTCATCAGGTGGATCATCAGGTGGATCT**TCAGAGGTGGAGTTCTCACATGAGTACTGGATGAGGCACGCTCTTACTTTGGCTAAGAGAGCTAGAGACGAGAGAGAAGTGCCAGTGGGTGCTGTTCTTGTTTTGAATAACAGAGTTATTGGAGAGGGATGGAATAGGGCTATCGGACTTCATGATCCAACAGCCCACGCCGAGATTATGGCCTTGAGGCAAGGTGGATTGGTTATGCAGAACTATAGGCTTATCGATGCTACTCTTTATGTTACATTTGAACCATGCGTGATGTGTGCCGGAGCCATGATCCACTCAAGGATTGGAAGGGTTGTTTTTGGAGTTAGAAATGCTAAAACTGGAGCCGCCGGTTCTCTTATGGATGTGTTGCATTATCCTGGTATGAACCATAGGGTTGAAATTACAGAGGGAATCTTGGCCGACGAGTGCGCTGCTCTTTTGTGCTACTTCTTTAGAATGCCAAGACAAGTTTTTAACGCCCAAAAGAAAGCCCAATCTTCAACCGAC**TCTGGTGGATCATCAGGTGGAAGTTCAGGTTCAGAGACCCCTGGAACTTCTGAATCAGCTACTCCAGAGTCCTCAGGTGGATCATCAGGTGGATCG**gacaagaagtactcgatcggcctcgccatcgggacgaactcagttggctgggccgtgatcaccgacgagtacaaggtgccctctaagaagttcaaggtcctggggaacaccgaccgccattccatcaagaagaacctcatcggcgctctcctgttcgacagcggggagaccgctgaggctacgaggctcaagagaaccgctaggcgccggtacacgagaaggaagaacaggatctgctacctccaagagattttctccaacgagatggccaaggttgacgattcattcttccaccgcctggaggagtctttcctcgtggaggaggataagaagcacgagcggcatcccatcttcggcaacatcgtggacgaggttgcctaccacgagaagtaccctacgatctaccatctgcggaagaagctcgtggactccaccgataaggcggacctcagactgatctacctcgctctggcccacatgatcaagttccgcggccatttcctgatcgagggggatctcaacccagacaacagcgatgttgacaagctgttcatccaactcgtgcagacctacaaccaactcttcgaggagaacccgatcaacgcctctggcgtggacgcgaaggctatcctgtccgcgaggctctcgaagtccaggaggctggagaacctgatcgctcagctcccaggcgagaagaagaacggcctgttcgggaacctcatcgctctcagcctggggctcaccccgaacttcaagtcgaacttcgatctcgctgaggacgccaagctgcaactctccaaggacacctacgacgatgacctcgataacctcctggcccagatcggcgatcaatacgcggacctgttcctcgctgccaagaacctgtcggacgccatcctcctgtcagatatcctccgcgtgaacaccgagatcacgaaggctccactctctgcctccatgatcaagcgctacgacgagcaccatcaggatctgaccctcctgaaggcgctggtccgccaacagctcccggagaagtacaaggagattttcttcgatcagtcgaagaacggctacgctgggtacatcgacggcggggcctcacaagaggagttctacaagttcatcaagccaatcctggagaagatggacggcacggaggagctcctggtgaagctcaacagggaggacctcctgcggaagcagagaaccttcgataacggcagcatcccccaccaaatccatctcggggagctgcacgccatcctgagaaggcaagaggacttctaccctttcctcaaggataaccgggagaagatcgagaagatcctgaccttcagaatcccatactacgtcggccctctcgcgcgggggaactcaagattcgcttggatgacccgcaagtctgaggagaccatcacgccgtggaacttcgaggaggtggtggacaagggcgctagcgctcagtcgttcatcgagaggatgaccaacttcgacaagaacctgcccaacgagaaggtgctccctaagcactcgctcctgtacgagtacttcaccgtctacaacgagctcacgaaggtgaagtacgtcaccgagggcatgcgcaagccagcgttcctgtccggggagcagaagaaggctatcgtggacctcctgttcaagaccaaccggaaggtcacggttaagcaactcaaggaggactacttcaagaagatcgagtgcttcgattcggtcgagatcagcggcgttgaggaccgcttcaacgccagcctcgggacctaccacgatctcctgaagatcatcaaggataaggacttcctggacaacgaggagaacgaggatatcctggaggacatcgtgctgaccctcacgctgttcgaggacagggagatgatcgaggagcgcctgaagacgtacgcccatctcttcgatgacaaggtcatgaagcaactcaagcgccggagatacaccggctgggggaggctgtcccgcaagctcatcaacggcatccgggacaagcagtccgggaagaccatcctcgacttcctcaagagcgatggcttcgccaacaggaacttcatgcaactgatccacgatgacagcctcaccttcaaggaggatatccaaaaggctcaagtgagcggccagggggactcgctgcacgagcatatcgcgaacctcgctggctcccccgcgatcaagaagggcatcctccagaccgtgaaggttgtggacgagctcgtgaaggtcatgggccggcacaagcctgagaacatcgtcatcgagatggccagagagaaccaaaccacgcagaaggggcaaaagaactctagggagcgcatgaagcgcatcgaggagggcatcaaggagctggggtcccaaatcctcaaggagcacccagtggagaacacccaactgcagaacgagaagctctacctgtactacctccagaacggcagggatatgtacgtggaccaagagctggatatcaaccgcctcagcgattacgacgtcgatcatatcgttccccagtctttcctgaaggatgactccatcgacaacaaggtcctcaccaggtcggacaagaaccgcggcaagtcagataacgttccatctgaggaggtcgttaagaagatgaagaactactggaggcagctcctgaacgccaagctgatcacgcaaaggaagttcgacaacctcaccaaggctgagagaggcgggctctcagagctggacaaggccggcttcatcaagcggcagctggtcgagaccagacaaatcacgaagcacgttgcgcaaatcctcgactctcggatgaacacgaagtacgatgagaacgacaagctgatcagggaggttaaggtgatcaccctgaagtctaagctcgtctccgacttcaggaaggatttccagttctacaaggttcgcgagatcaacaactaccaccatgcccatgacgcttacctcaacgctgtggtcggcaccgctctgatcaagaagtacccaaagctggagtccgagttcgtgtacggggactacaaggtttacgatgtgcgcaagatgatcgccaagtcggagcaagagatcggcaaggctaccgccaagtacttcttctactcaaacatcatgaacttcttcaagaccgagatcacgctggccaacggcgagatccggaagagaccgctcatcgagaccaacggcgagacgggggagatcgtgtgggacaagggcagggatttcgcgaccgtccgcaaggttctctccatgccccaggtgaacatcgtcaagaagaccgaggtccaaacgggcgggttctcaaaggagtctatcctgcctaagcggaacagcgacaagctcatcgccagaaagaaggactgggacccaaagaagtacggcgggttcgacagccctaccgtggcctactcggtcctggttgtggcgaaggttgagaagggcaagtccaagaagctcaagagcgtgaaggagctcctggggatcaccatcatggagaggtccagcttcgagaagaacccaatcgacttcctggaggccaagggctacaaggaggtgaagaaggacctgatcatcaagctcccgaagtactctctcttcgagctggagaacggcaggaagagaatgctggcttccgctggcgagctccagaaggggaacgagctcgcgctgccaagcaagtacgtgaacttcctctacctggcttcccactacgagaagctcaagggcagcccggaggacaacgagcaaaagcagctgttcgtcgagcagcacaagcattacctcgacgagatcatcgagcaaatctccgagttcagcaagcgcgtgatcctcgccgacgcgaacctggataaggtcctctccgcctacaacaagcaccgggacaagcccatcagagagcaagcggagaacatcatccatctcttcaccctgacgaacctcggcgctcctgctgctttcaagtacttcgacaccacgatcgatcggaagagatacacctccacgaaggaggtcctggacgcgaccctcatccaccagtcgatcaccggcctgtacgagacgaggatcgacctctcacaactcggcggggataagagacccgcagcaaccaagaaggcagggcaagcaaagaagaagaagacgcgt**ccaaagaagaagcggaaggtg

### GhABE6.3dCas9

(NLS-TadA(wt)–linker(32 aa)–TadA*(6.3)–linker(32 aa)–dCas9–NLS

**ATG**ccaaagaagaagaggaaggtt**TCTGAAGTTGAGTTCTCACATGAATATTGGATGAGACATGCTCTTACATTGGCTAAGAGAGCTTGGGATGAAAGGGAGGTTCCTGTTGGTGCTGTTCTTGTTCATAATAACAGGGTTATTGGTGAAGGATGGAATAGACCTATCGGAAGGCATGATCCAACCGCTCATGCTGAGATTATGGCTCTTAGACAAGGTGGATTGGTTATGCAAAACTATAGGTTGATCGATGCTACACTTTACGTTACCTTGGAGCCTTGTGTTATGTGCGCTGGTGCTATGATTCATTCTAGAATCGGTAGGGTTGTTTTTGGAGCTAGAGATGCTAAGACCGGTGCTGCTGGATCACTTATGGATGTTTTGCATCATCCTGGTATGAATCATAGAGTTGAAATTACTGAGGGAATCCTTGCTGATGAATGTGCTGCTCTTTTGTCTGATTTCTTTAGGATGAGAAGGCAAGAGATTAAAGCTCAAAAGAAAGCTCAATCTTCAACAGATTCTGGTGGATCTTCAGGTGGATCTTCAGGTTCAGAAACTCCTGGAACATCTGAATCAGCTACCCCAGAGTCATCAGGTGGATCATCAGGTGGATCTTCAGAAGTTGAGTTCTCTCATGAGTACTGGATGAGGCACGCTCTTACTCTTGCCAAAAGGGCCTGGGACGAGAGAGAAGTGCCAGTGGGTGCTGTTCTTGTTTTGAATAACAGAGTTATTGGAGAGGGATGGAATAGGTCAATCGGACTTCATGATCCAACAGCCCACGCCGAGATTATGGCCTTGAGGCAAGGTGGATTGGTTATGCAGAACTATAGGCTTATCGATGCTACTCTTTATGTTACATTTGAACCATGCGTGATGTGTGCCGGAGCCATGATCCACTCAAGGATTGGAAGAGTTGTTTTTGGAGTTAGGAATGCTAAAACTGGAGCCGCCGGTTCTCTTATGGATGTGTTGCATTATCCTGGTATGAACCATAGAGTTGAAATTACAGAGGGAATCTTGGCCGACGAGTGCGCTGCTCTTTTGTGCTACTTCTTTAGAATGAGAAGGCAGGTTTTTAACGCCCAAAAGAAAGCCCAATCTTCAACCGACTCTGGTGGATCATCAGGTGGAAGTTCAGGTTCAGAGACCCCTGGAACTTCTGAATCAGCTACTCCAGAGTCCTCAGGTGGATCATCAGGTGGATCGgacaagaagtactcgatcggcctcgccatcgggacgaactcagttggctgggccgtgatcaccgacgagtacaaggtgccctctaagaagttcaaggtcctggggaacaccgaccgccattccatcaagaagaacctcatcggcgctctcctgttcgacagcggggagaccgctgaggctacgaggctcaagagaaccgctaggcgccggtacacgagaaggaagaacaggatctgctacctccaagagattttctccaacgagatggccaaggttgacgattcattcttccaccgcctggaggagtctttcctcgtggaggaggataagaagcacgagcggcatcccatcttcggcaacatcgtggacgaggttgcctaccacgagaagtaccctacgatctaccatctgcggaagaagctcgtggactccaccgataaggcggacctcagactgatctacctcgctctggcccacatgatcaagttccgcggccatttcctgatcgagggggatctcaacccagacaacagcgatgttgacaagctgttcatccaactcgtgcagacctacaaccaactcttcgaggagaacccgatcaacgcctctggcgtggacgcgaaggctatcctgtccgcgaggctctcgaagtccaggaggctggagaacctgatcgctcagctcccaggcgagaagaagaacggcctgttcgggaacctcatcgctctcagcctggggctcaccccgaacttcaagtcgaacttcgatctcgctgaggacgccaagctgcaactctccaaggacacctacgacgatgacctcgataacctcctggcccagatcggcgatcaatacgcggacctgttcctcgctgccaagaacctgtcggacgccatcctcctgtcagatatcctccgcgtgaacaccgagatcacgaaggctccactctctgcctccatgatcaagcgctacgacgagcaccatcaggatctgaccctcctgaaggcgctggtccgccaacagctcccggagaagtacaaggagattttcttcgatcagtcgaagaacggctacgctgggtacatcgacggcggggcctcacaagaggagttctacaagttcatcaagccaatcctggagaagatggacggcacggaggagctcctggtgaagctcaacagggaggacctcctgcggaagcagagaaccttcgataacggcagcatcccccaccaaatccatctcggggagctgcacgccatcctgagaaggcaagaggacttctaccctttcctcaaggataaccgggagaagatcgagaagatcctgaccttcagaatcccatactacgtcggccctctcgcgcgggggaactcaagattcgcttggatgacccgcaagtctgaggagaccatcacgccgtggaacttcgaggaggtggtggacaagggcgctagcgctcagtcgttcatcgagaggatgaccaacttcgacaagaacctgcccaacgagaaggtgctccctaagcactcgctcctgtacgagtacttcaccgtctacaacgagctcacgaaggtgaagtacgtcaccgagggcatgcgcaagccagcgttcctgtccggggagcagaagaaggctatcgtggacctcctgttcaagaccaaccggaaggtcacggttaagcaactcaaggaggactacttcaagaagatcgagtgcttcgattcggtcgagatcagcggcgttgaggaccgcttcaacgccagcctcgggacctaccacgatctcctgaagatcatcaaggataaggacttcctggacaacgaggagaacgaggatatcctggaggacatcgtgctgaccctcacgctgttcgaggacagggagatgatcgaggagcgcctgaagacgtacgcccatctcttcgatgacaaggtcatgaagcaactcaagcgccggagatacaccggctgggggaggctgtcccgcaagctcatcaacggcatccgggacaagcagtccgggaagaccatcctcgacttcctcaagagcgatggcttcgccaacaggaacttcatgcaactgatccacgatgacagcctcaccttcaaggaggatatccaaaaggctcaagtgagcggccagggggactcgctgcacgagcatatcgcgaacctcgctggctcccccgcgatcaagaagggcatcctccagaccgtgaaggttgtggacgagctcgtgaaggtcatgggccggcacaagcctgagaacatcgtcatcgagatggccagagagaaccaaaccacgcagaaggggcaaaagaactctagggagcgcatgaagcgcatcgaggagggcatcaaggagctggggtcccaaatcctcaaggagcacccagtggagaacacccaactgcagaacgagaagctctacctgtactacctccagaacggcagggatatgtacgtggaccaagagctggatatcaaccgcctcagcgattacgacgtcgatgctatcgttccccagtctttcctgaaggatgactccatcgacaacaaggtcctcaccaggtcggacaagaaccgcggcaagtcagataacgttccatctgaggaggtcgttaagaagatgaagaactactggaggcagctcctgaacgccaagctgatcacgcaaaggaagttcgacaacctcaccaaggctgagagaggcgggctctcagagctggacaaggccggcttcatcaagcggcagctggtcgagaccagacaaatcacgaagcacgttgcgcaaatcctcgactctcggatgaacacgaagtacgatgagaacgacaagctgatcagggaggttaaggtgatcaccctgaagtctaagctcgtctccgacttcaggaaggatttccagttctacaaggttcgcgagatcaacaactaccaccatgcccatgacgcttacctcaacgctgtggtcggcaccgctctgatcaagaagtacccaaagctggagtccgagttcgtgtacggggactacaaggtttacgatgtgcgcaagatgatcgccaagtcggagcaagagatcggcaaggctaccgccaagtacttcttctactcaaacatcatgaacttcttcaagaccgagatcacgctggccaacggcgagatccggaagagaccgctcatcgagaccaacggcgagacgggggagatcgtgtgggacaagggcagggatttcgcgaccgtccgcaaggttctctccatgccccaggtgaacatcgtcaagaagaccgaggtccaaacgggcgggttctcaaaggagtctatcctgcctaagcggaacagcgacaagctcatcgccagaaagaaggactgggacccaaagaagtacggcgggttcgacagccctaccgtggcctactcggtcctggttgtggcgaaggttgagaagggcaagtccaagaagctcaagagcgtgaaggagctcctggggatcaccatcatggagaggtccagcttcgagaagaacccaatcgacttcctggaggccaagggctacaaggaggtgaagaaggacctgatcatcaagctcccgaagtactctctcttcgagctggagaacggcaggaagagaatgctggcttccgctggcgagctccagaaggggaacgagctcgcgctgccaagcaagtacgtgaacttcctctacctggcttcccactacgagaagctcaagggcagcccggaggacaacgagcaaaagcagctgttcgtcgagcagcacaagcattacctcgacgagatcatcgagcaaatctccgagttcagcaagcgcgtgatcctcgccgacgcgaacctggataaggtcctctccgcctacaacaagcaccgggacaagcccatcagagagcaagcggagaacatcatccatctcttcaccctgacgaacctcggcgctcctgctgctttcaagtacttcgacaccacgatcgatcggaagagatacacctccacgaaggaggtcctggacgcgaccctcatccaccagtcgatcaccggcctgtacgagacgaggatcgacctctcacaactcggcggggataagagacccgcagcaaccaagaaggcagggcaagcaaagaagaagaagacgcgt**ccaaagaagaagcggaaggtg

### GhABE7.8dCas9

(NLS-TadA(wt)–linker(32 aa)–TadA*(7.8)–linker(32 aa)–dCas9–NLS

**ATG**ccaaagaagaagaggaaggtt**TCTGAAGTTGAGTTCTCACATGAATATTGGATGAGACATGCTCTTACATTGGCTAAGAGAGCTTGGGATGAAAGGGAGGTTCCTGTTGGTGCTGTTCTTGTTCATAATAACAGGGTTATTGGTGAAGGATGGAACAGACCTATCGGAAGGCATGATCCAACCGCTCATGCTGAGATTATGGCTCTTAGACAAGGTGGATTGGTTATGCAAAATTATAGGTTGATCGATGCTACACTTTACGTTACCTTGGAGCCTTGTGTTATGTGCGCTGGTGCTATGATTCATTCTAGAATCGGTAGGGTTGTTTTTGGAGCTAGAGATGCTAAGACCGGTGCTGCTGGATCACTTATGGATGTTTTGCATCATCCTGGTATGAACCATAGAGTTGAAATTACTGAGGGAATCCTTGCTGATGAATGTGCTGCTCTTTTGTCTGATTTCTTTAGGATGAGAAGGCAAGAGATTAAAGCTCAAAAGAAAGCTCAATCTTCAACAGAT**TCTGGTGGATCTTCAGGTGGATCTTCAGGTTCAGAAACTCCTGGAACATCTGAATCAGCTACCCCAGAGTCATCAGGTGGATCATCAGGTGGATCT**TCAGAGGTGGAGTTCTCACATGAGTACTGGATGAGGCACGCTCTTACTTTGGCTAAGAGAGCTCTTGACGAGAGAGAAGTGCCAGTGGGAGCTGTTCTTGTTTTGAATAACAGAGTTATTGGAGAGGGATGGAACAGGGCTATCGGATTGCATGATCCAACAGCCCACGCCGAGATTATGGCCTTGAGGCAAGGTGGATTGGTTATGCAGAATTATAGGCTTATCGATGCTACTCTTTATGTTACATTTGAACCATGCGTGATGTGTGCCGGAGCCATGATCCACTCAAGGATTGGAAGAGTTGTTTTTGGAGTTAGGAATGCTAAAACTGGAGCCGCCGGTTCTCTTATGGATGTGTTGCATTATCCTGGTATGAATCATAGAGTTGAAATTACAGAGGGAATCTTGGCTGATGAATGTAACGCTCTTTTGTGCTACTTCTTTAGAATGAGAAGGCAGGTTTTTAATGCCCAAAAGAAAGCCCAATCTTCAACCGAC**TCTGGTGGATCATCAGGTGGAAGTTCAGGTTCAGAGACCCCTGGAACTTCTGAATCAGCTACTCCAGAGTCCTCAGGTGGATCATCAGGTGGATCG**gacaagaagtactcgatcggcctcgccatcgggacgaactcagttggctgggccgtgatcaccgacgagtacaaggtgccctctaagaagttcaaggtcctggggaacaccgaccgccattccatcaagaagaacctcatcggcgctctcctgttcgacagcggggagaccgctgaggctacgaggctcaagagaaccgctaggcgccggtacacgagaaggaagaacaggatctgctacctccaagagattttctccaacgagatggccaaggttgacgattcattcttccaccgcctggaggagtctttcctcgtggaggaggataagaagcacgagcggcatcccatcttcggcaacatcgtggacgaggttgcctaccacgagaagtaccctacgatctaccatctgcggaagaagctcgtggactccaccgataaggcggacctcagactgatctacctcgctctggcccacatgatcaagttccgcggccatttcctgatcgagggggatctcaacccagacaacagcgatgttgacaagctgttcatccaactcgtgcagacctacaaccaactcttcgaggagaacccgatcaacgcctctggcgtggacgcgaaggctatcctgtccgcgaggctctcgaagtccaggaggctggagaacctgatcgctcagctcccaggcgagaagaagaacggcctgttcgggaacctcatcgctctcagcctggggctcaccccgaacttcaagtcgaacttcgatctcgctgaggacgccaagctgcaactctccaaggacacctacgacgatgacctcgataacctcctggcccagatcggcgatcaatacgcggacctgttcctcgctgccaagaacctgtcggacgccatcctcctgtcagatatcctccgcgtgaacaccgagatcacgaaggctccactctctgcctccatgatcaagcgctacgacgagcaccatcaggatctgaccctcctgaaggcgctggtccgccaacagctcccggagaagtacaaggagattttcttcgatcagtcgaagaacggctacgctgggtacatcgacggcggggcctcacaagaggagttctacaagttcatcaagccaatcctggagaagatggacggcacggaggagctcctggtgaagctcaacagggaggacctcctgcggaagcagagaaccttcgataacggcagcatcccccaccaaatccatctcggggagctgcacgccatcctgagaaggcaagaggacttctaccctttcctcaaggataaccgggagaagatcgagaagatcctgaccttcagaatcccatactacgtcggccctctcgcgcgggggaactcaagattcgcttggatgacccgcaagtctgaggagaccatcacgccgtggaacttcgaggaggtggtggacaagggcgctagcgctcagtcgttcatcgagaggatgaccaacttcgacaagaacctgcccaacgagaaggtgctccctaagcactcgctcctgtacgagtacttcaccgtctacaacgagctcacgaaggtgaagtacgtcaccgagggcatgcgcaagccagcgttcctgtccggggagcagaagaaggctatcgtggacctcctgttcaagaccaaccggaaggtcacggttaagcaactcaaggaggactacttcaagaagatcgagtgcttcgattcggtcgagatcagcggcgttgaggaccgcttcaacgccagcctcgggacctaccacgatctcctgaagatcatcaaggataaggacttcctggacaacgaggagaacgaggatatcctggaggacatcgtgctgaccctcacgctgttcgaggacagggagatgatcgaggagcgcctgaagacgtacgcccatctcttcgatgacaaggtcatgaagcaactcaagcgccggagatacaccggctgggggaggctgtcccgcaagctcatcaacggcatccgggacaagcagtccgggaagaccatcctcgacttcctcaagagcgatggcttcgccaacaggaacttcatgcaactgatccacgatgacagcctcaccttcaaggaggatatccaaaaggctcaagtgagcggccagggggactcgctgcacgagcatatcgcgaacctcgctggctcccccgcgatcaagaagggcatcctccagaccgtgaaggttgtggacgagctcgtgaaggtcatgggccggcacaagcctgagaacatcgtcatcgagatggccagagagaaccaaaccacgcagaaggggcaaaagaactctagggagcgcatgaagcgcatcgaggagggcatcaaggagctggggtcccaaatcctcaaggagcacccagtggagaacacccaactgcagaacgagaagctctacctgtactacctccagaacggcagggatatgtacgtggaccaagagctggatatcaaccgcctcagcgattacgacgtcgatgctatcgttccccagtctttcctgaaggatgactccatcgacaacaaggtcctcaccaggtcggacaagaaccgcggcaagtcagataacgttccatctgaggaggtcgttaagaagatgaagaactactggaggcagctcctgaacgccaagctgatcacgcaaaggaagttcgacaacctcaccaaggctgagagaggcgggctctcagagctggacaaggccggcttcatcaagcggcagctggtcgagaccagacaaatcacgaagcacgttgcgcaaatcctcgactctcggatgaacacgaagtacgatgagaacgacaagctgatcagggaggttaaggtgatcaccctgaagtctaagctcgtctccgacttcaggaaggatttccagttctacaaggttcgcgagatcaacaactaccaccatgcccatgacgcttacctcaacgctgtggtcggcaccgctctgatcaagaagtacccaaagctggagtccgagttcgtgtacggggactacaaggtttacgatgtgcgcaagatgatcgccaagtcggagcaagagatcggcaaggctaccgccaagtacttcttctactcaaacatcatgaacttcttcaagaccgagatcacgctggccaacggcgagatccggaagagaccgctcatcgagaccaacggcgagacgggggagatcgtgtgggacaagggcagggatttcgcgaccgtccgcaaggttctctccatgccccaggtgaacatcgtcaagaagaccgaggtccaaacgggcgggttctcaaaggagtctatcctgcctaagcggaacagcgacaagctcatcgccagaaagaaggactgggacccaaagaagtacggcgggttcgacagccctaccgtggcctactcggtcctggttgtggcgaaggttgagaagggcaagtccaagaagctcaagagcgtgaaggagctcctggggatcaccatcatggagaggtccagcttcgagaagaacccaatcgacttcctggaggccaagggctacaaggaggtgaagaaggacctgatcatcaagctcccgaagtactctctcttcgagctggagaacggcaggaagagaatgctggcttccgctggcgagctccagaaggggaacgagctcgcgctgccaagcaagtacgtgaacttcctctacctggcttcccactacgagaagctcaagggcagcccggaggacaacgagcaaaagcagctgttcgtcgagcagcacaagcattacctcgacgagatcatcgagcaaatctccgagttcagcaagcgcgtgatcctcgccgacgcgaacctggataaggtcctctccgcctacaacaagcaccgggacaagcccatcagagagcaagcggagaacatcatccatctcttcaccctgacgaacctcggcgctcctgctgctttcaagtacttcgacaccacgatcgatcggaagagatacacctccacgaaggaggtcctggacgcgaccctcatccaccagtcgatcaccggcctgtacgagacgaggatcgacctctcacaactcggcggggataagagacccgcagcaaccaagaaggcagggcaagcaaagaagaagaagacgcgt**ccaaagaagaagcggaaggtg

### GhABE7.9dCas9

(NLS-TadA(wt)–linker(32 aa)–TadA*(7.9)–linker(32 aa)–dCas9–NLS

**ATG**ccaaagaagaagaggaaggtt**TCTGAAGTTGAGTTCTCACATGAATATTGGATGAGACATGCTCTTACATTGGCTAAGAGAGCTTGGGATGAAAGGGAGGTTCCTGTTGGTGCTGTTCTTGTTCATAATAACAGGGTTATTGGTGAAGGATGGAACAGACCTATCGGAAGGCATGATCCAACCGCTCATGCTGAGATTATGGCTCTTAGACAAGGTGGATTGGTTATGCAAAATTATAGGTTGATCGATGCTACACTTTACGTTACCTTGGAGCCTTGTGTTATGTGCGCTGGTGCTATGATTCATTCTAGAATCGGTAGGGTTGTTTTTGGAGCTAGAGATGCTAAGACCGGTGCTGCTGGATCACTTATGGATGTTTTGCATCATCCTGGTATGAACCATAGAGTTGAAATTACTGAGGGAATCCTTGCTGATGAATGTGCTGCTCTTTTGTCTGATTTCTTTAGGATGAGAAGGCAAGAGATTAAAGCTCAAAAGAAAGCTCAATCTTCAACAGAT**TCTGGTGGATCTTCAGGTGGATCTTCAGGTTCAGAAACTCCTGGAACATCTGAATCAGCTACCCCAGAGTCATCAGGTGGATCATCAGGTGGATCT**TCAGAGGTGGAGTTCTCACATGAGTACTGGATGAGGCATGCTCTTACTTTGGCTAAGAGAGCTCTTGACGAGAGAGAAGTGCCAGTGGGAGCTGTTCTTGTTTTGAATAACAGAGTTATTGGAGAGGGATGGAACAGGGCTATCGGATTGCATGATCCAACAGCCCACGCCGAGATTATGGCCTTGAGGCAAGGTGGATTGGTTATGCAGAATTATAGGCTTATCGATGCTACTCTTTATGTTACATTTGAACCATGCGTGATGTGTGCCGGAGCCATGATCCACTCAAGGATTGGAAGGGTTGTTTTTGGAGTTAGAAATGCTAAAACTGGAGCCGCCGGTTCTCTTATGGATGTGTTGCATTATCCTGGTATGAATCATAGGGTTGAAATTACAGAGGGAATCTTGGCTGATGAATGTAACGCTCTTTTGTGCTACTTCTTTAGAATGCCAAGACAAGTTTTTAATGCCCAAAAGAAAGCCCAATCTTCAACCGAC**TCTGGTGGATCATCAGGTGGAAGTTCAGGTTCAGAGACCCCTGGAACTTCTGAATCAGCTACTCCAGAGTCCTCAGGTGGATCATCAGGTGGATCG**gacaagaagtactcgatcggcctcgccatcgggacgaactcagttggctgggccgtgatcaccgacgagtacaaggtgccctctaagaagttcaaggtcctggggaacaccgaccgccattccatcaagaagaacctcatcggcgctctcctgttcgacagcggggagaccgctgaggctacgaggctcaagagaaccgctaggcgccggtacacgagaaggaagaacaggatctgctacctccaagagattttctccaacgagatggccaaggttgacgattcattcttccaccgcctggaggagtctttcctcgtggaggaggataagaagcacgagcggcatcccatcttcggcaacatcgtggacgaggttgcctaccacgagaagtaccctacgatctaccatctgcggaagaagctcgtggactccaccgataaggcggacctcagactgatctacctcgctctggcccacatgatcaagttccgcggccatttcctgatcgagggggatctcaacccagacaacagcgatgttgacaagctgttcatccaactcgtgcagacctacaaccaactcttcgaggagaacccgatcaacgcctctggcgtggacgcgaaggctatcctgtccgcgaggctctcgaagtccaggaggctggagaacctgatcgctcagctcccaggcgagaagaagaacggcctgttcgggaacctcatcgctctcagcctggggctcaccccgaacttcaagtcgaacttcgatctcgctgaggacgccaagctgcaactctccaaggacacctacgacgatgacctcgataacctcctggcccagatcggcgatcaatacgcggacctgttcctcgctgccaagaacctgtcggacgccatcctcctgtcagatatcctccgcgtgaacaccgagatcacgaaggctccactctctgcctccatgatcaagcgctacgacgagcaccatcaggatctgaccctcctgaaggcgctggtccgccaacagctcccggagaagtacaaggagattttcttcgatcagtcgaagaacggctacgctgggtacatcgacggcggggcctcacaagaggagttctacaagttcatcaagccaatcctggagaagatggacggcacggaggagctcctggtgaagctcaacagggaggacctcctgcggaagcagagaaccttcgataacggcagcatcccccaccaaatccatctcggggagctgcacgccatcctgagaaggcaagaggacttctaccctttcctcaaggataaccgggagaagatcgagaagatcctgaccttcagaatcccatactacgtcggccctctcgcgcgggggaactcaagattcgcttggatgacccgcaagtctgaggagaccatcacgccgtggaacttcgaggaggtggtggacaagggcgctagcgctcagtcgttcatcgagaggatgaccaacttcgacaagaacctgcccaacgagaaggtgctccctaagcactcgctcctgtacgagtacttcaccgtctacaacgagctcacgaaggtgaagtacgtcaccgagggcatgcgcaagccagcgttcctgtccggggagcagaagaaggctatcgtggacctcctgttcaagaccaaccggaaggtcacggttaagcaactcaaggaggactacttcaagaagatcgagtgcttcgattcggtcgagatcagcggcgttgaggaccgcttcaacgccagcctcgggacctaccacgatctcctgaagatcatcaaggataaggacttcctggacaacgaggagaacgaggatatcctggaggacatcgtgctgaccctcacgctgttcgaggacagggagatgatcgaggagcgcctgaagacgtacgcccatctcttcgatgacaaggtcatgaagcaactcaagcgccggagatacaccggctgggggaggctgtcccgcaagctcatcaacggcatccgggacaagcagtccgggaagaccatcctcgacttcctcaagagcgatggcttcgccaacaggaacttcatgcaactgatccacgatgacagcctcaccttcaaggaggatatccaaaaggctcaagtgagcggccagggggactcgctgcacgagcatatcgcgaacctcgctggctcccccgcgatcaagaagggcatcctccagaccgtgaaggttgtggacgagctcgtgaaggtcatgggccggcacaagcctgagaacatcgtcatcgagatggccagagagaaccaaaccacgcagaaggggcaaaagaactctagggagcgcatgaagcgcatcgaggagggcatcaaggagctggggtcccaaatcctcaaggagcacccagtggagaacacccaactgcagaacgagaagctctacctgtactacctccagaacggcagggatatgtacgtggaccaagagctggatatcaaccgcctcagcgattacgacgtcgatgctatcgttccccagtctttcctgaaggatgactccatcgacaacaaggtcctcaccaggtcggacaagaaccgcggcaagtcagataacgttccatctgaggaggtcgttaagaagatgaagaactactggaggcagctcctgaacgccaagctgatcacgcaaaggaagttcgacaacctcaccaaggctgagagaggcgggctctcagagctggacaaggccggcttcatcaagcggcagctggtcgagaccagacaaatcacgaagcacgttgcgcaaatcctcgactctcggatgaacacgaagtacgatgagaacgacaagctgatcagggaggttaaggtgatcaccctgaagtctaagctcgtctccgacttcaggaaggatttccagttctacaaggttcgcgagatcaacaactaccaccatgcccatgacgcttacctcaacgctgtggtcggcaccgctctgatcaagaagtacccaaagctggagtccgagttcgtgtacggggactacaaggtttacgatgtgcgcaagatgatcgccaagtcggagcaagagatcggcaaggctaccgccaagtacttcttctactcaaacatcatgaacttcttcaagaccgagatcacgctggccaacggcgagatccggaagagaccgctcatcgagaccaacggcgagacgggggagatcgtgtgggacaagggcagggatttcgcgaccgtccgcaaggttctctccatgccccaggtgaacatcgtcaagaagaccgaggtccaaacgggcgggttctcaaaggagtctatcctgcctaagcggaacagcgacaagctcatcgccagaaagaaggactgggacccaaagaagtacggcgggttcgacagccctaccgtggcctactcggtcctggttgtggcgaaggttgagaagggcaagtccaagaagctcaagagcgtgaaggagctcctggggatcaccatcatggagaggtccagcttcgagaagaacccaatcgacttcctggaggccaagggctacaaggaggtgaagaaggacctgatcatcaagctcccgaagtactctctcttcgagctggagaacggcaggaagagaatgctggcttccgctggcgagctccagaaggggaacgagctcgcgctgccaagcaagtacgtgaacttcctctacctggcttcccactacgagaagctcaagggcagcccggaggacaacgagcaaaagcagctgttcgtcgagcagcacaagcattacctcgacgagatcatcgagcaaatctccgagttcagcaagcgcgtgatcctcgccgacgcgaacctggataaggtcctctccgcctacaacaagcaccgggacaagcccatcagagagcaagcggagaacatcatccatctcttcaccctgacgaacctcggcgctcctgctgctttcaagtacttcgacaccacgatcgatcggaagagatacacctccacgaaggaggtcctggacgcgaccctcatccaccagtcgatcaccggcctgtacgagacgaggatcgacctctcacaactcggcggggataagagacccgcagcaaccaagaaggcagggcaagcaaagaagaagaagacgcgt**ccaaagaagaagcggaaggtg

### GhABE7.10dCas9

(NLS-TadA(wt)–linker(32 aa)–TadA*(7.10)–linker(32 aa)–dCas9–NLS

**ATG**ccaaagaagaagaggaaggtt**TCTGAAGTTGAGTTCTCACATGAATATTGGATGAGACATGCTCTTACATTGGCTAAGAGAGCTTGGGATGAAAGGGAGGTTCCTGTTGGTGCTGTTCTTGTTCATAATAACAGGGTTATTGGTGAAGGATGGAATAGACCTATCGGAAGGCATGATCCAACCGCTCATGCTGAGATTATGGCTCTTAGACAAGGTGGATTGGTTATGCAAAACTATAGGTTGATCGATGCTACACTTTACGTTACCTTGGAGCCTTGTGTTATGTGCGCTGGTGCTATGATTCATTCTAGAATCGGTAGGGTTGTTTTTGGAGCTAGAGATGCTAAGACCGGTGCTGCTGGATCACTTATGGATGTTTTGCATCATCCTGGTATGAATCATAGAGTTGAAATTACTGAGGGAATCCTTGCTGATGAATGTGCTGCTCTTTTGTCTGATTTCTTTAGGATGAGAAGGCAAGAGATTAAAGCTCAAAAGAAAGCTCAATCTTCAACAGAT**TCTGGTGGATCTTCAGGTGGATCTTCAGGTTCAGAAACTCCTGGAACATCTGAATCAGCTACCCCAGAGTCATCAGGTGGATCATCAGGTGGATCT**TCAGAGGTGGAGTTCTCACATGAGTACTGGATGAGGCACGCTCTTACTTTGGCTAAGAGAGCTAGAGACGAGAGAGAAGTGCCAGTGGGTGCTGTTCTTGTTTTGAATAACAGAGTTATTGGAGAGGGATGGAATAGGGCTATCGGACTTCATGATCCAACAGCCCACGCCGAGATTATGGCCTTGAGGCAAGGTGGATTGGTTATGCAGAACTATAGGCTTATCGATGCTACTCTTTATGTTACATTTGAACCATGCGTGATGTGTGCCGGAGCCATGATCCACTCAAGGATTGGAAGGGTTGTTTTTGGAGTTAGAAATGCTAAAACTGGAGCCGCCGGTTCTCTTATGGATGTGTTGCATTATCCTGGTATGAACCATAGGGTTGAAATTACAGAGGGAATCTTGGCCGACGAGTGCGCTGCTCTTTTGTGCTACTTCTTTAGAATGCCAAGACAAGTTTTTAACGCCCAAAAGAAAGCCCAATCTTCAACCGAC**TCTGGTGGATCATCAGGTGGAAGTTCAGGTTCAGAGACCCCTGGAACTTCTGAATCAGCTACTCCAGAGTCCTCAGGTGGATCATCAGGTGGATCG**gacaagaagtactcgatcggcctcgccatcgggacgaactcagttggctgggccgtgatcaccgacgagtacaaggtgccctctaagaagttcaaggtcctggggaacaccgaccgccattccatcaagaagaacctcatcggcgctctcctgttcgacagcggggagaccgctgaggctacgaggctcaagagaaccgctaggcgccggtacacgagaaggaagaacaggatctgctacctccaagagattttctccaacgagatggccaaggttgacgattcattcttccaccgcctggaggagtctttcctcgtggaggaggataagaagcacgagcggcatcccatcttcggcaacatcgtggacgaggttgcctaccacgagaagtaccctacgatctaccatctgcggaagaagctcgtggactccaccgataaggcggacctcagactgatctacctcgctctggcccacatgatcaagttccgcggccatttcctgatcgagggggatctcaacccagacaacagcgatgttgacaagctgttcatccaactcgtgcagacctacaaccaactcttcgaggagaacccgatcaacgcctctggcgtggacgcgaaggctatcctgtccgcgaggctctcgaagtccaggaggctggagaacctgatcgctcagctcccaggcgagaagaagaacggcctgttcgggaacctcatcgctctcagcctggggctcaccccgaacttcaagtcgaacttcgatctcgctgaggacgccaagctgcaactctccaaggacacctacgacgatgacctcgataacctcctggcccagatcggcgatcaatacgcggacctgttcctcgctgccaagaacctgtcggacgccatcctcctgtcagatatcctccgcgtgaacaccgagatcacgaaggctccactctctgcctccatgatcaagcgctacgacgagcaccatcaggatctgaccctcctgaaggcgctggtccgccaacagctcccggagaagtacaaggagattttcttcgatcagtcgaagaacggctacgctgggtacatcgacggcggggcctcacaagaggagttctacaagttcatcaagccaatcctggagaagatggacggcacggaggagctcctggtgaagctcaacagggaggacctcctgcggaagcagagaaccttcgataacggcagcatcccccaccaaatccatctcggggagctgcacgccatcctgagaaggcaagaggacttctaccctttcctcaaggataaccgggagaagatcgagaagatcctgaccttcagaatcccatactacgtcggccctctcgcgcgggggaactcaagattcgcttggatgacccgcaagtctgaggagaccatcacgccgtggaacttcgaggaggtggtggacaagggcgctagcgctcagtcgttcatcgagaggatgaccaacttcgacaagaacctgcccaacgagaaggtgctccctaagcactcgctcctgtacgagtacttcaccgtctacaacgagctcacgaaggtgaagtacgtcaccgagggcatgcgcaagccagcgttcctgtccggggagcagaagaaggctatcgtggacctcctgttcaagaccaaccggaaggtcacggttaagcaactcaaggaggactacttcaagaagatcgagtgcttcgattcggtcgagatcagcggcgttgaggaccgcttcaacgccagcctcgggacctaccacgatctcctgaagatcatcaaggataaggacttcctggacaacgaggagaacgaggatatcctggaggacatcgtgctgaccctcacgctgttcgaggacagggagatgatcgaggagcgcctgaagacgtacgcccatctcttcgatgacaaggtcatgaagcaactcaagcgccggagatacaccggctgggggaggctgtcccgcaagctcatcaacggcatccgggacaagcagtccgggaagaccatcctcgacttcctcaagagcgatggcttcgccaacaggaacttcatgcaactgatccacgatgacagcctcaccttcaaggaggatatccaaaaggctcaagtgagcggccagggggactcgctgcacgagcatatcgcgaacctcgctggctcccccgcgatcaagaagggcatcctccagaccgtgaaggttgtggacgagctcgtgaaggtcatgggccggcacaagcctgagaacatcgtcatcgagatggccagagagaaccaaaccacgcagaaggggcaaaagaactctagggagcgcatgaagcgcatcgaggagggcatcaaggagctggggtcccaaatcctcaaggagcacccagtggagaacacccaactgcagaacgagaagctctacctgtactacctccagaacggcagggatatgtacgtggaccaagagctggatatcaaccgcctcagcgattacgacgtcgatgctatcgttccccagtctttcctgaaggatgactccatcgacaacaaggtcctcaccaggtcggacaagaaccgcggcaagtcagataacgttccatctgaggaggtcgttaagaagatgaagaactactggaggcagctcctgaacgccaagctgatcacgcaaaggaagttcgacaacctcaccaaggctgagagaggcgggctctcagagctggacaaggccggcttcatcaagcggcagctggtcgagaccagacaaatcacgaagcacgttgcgcaaatcctcgactctcggatgaacacgaagtacgatgagaacgacaagctgatcagggaggttaaggtgatcaccctgaagtctaagctcgtctccgacttcaggaaggatttccagttctacaaggttcgcgagatcaacaactaccaccatgcccatgacgcttacctcaacgctgtggtcggcaccgctctgatcaagaagtacccaaagctggagtccgagttcgtgtacggggactacaaggtttacgatgtgcgcaagatgatcgccaagtcggagcaagagatcggcaaggctaccgccaagtacttcttctactcaaacatcatgaacttcttcaagaccgagatcacgctggccaacggcgagatccggaagagaccgctcatcgagaccaacggcgagacgggggagatcgtgtgggacaagggcagggatttcgcgaccgtccgcaaggttctctccatgccccaggtgaacatcgtcaagaagaccgaggtccaaacgggcgggttctcaaaggagtctatcctgcctaagcggaacagcgacaagctcatcgccagaaagaaggactgggacccaaagaagtacggcgggttcgacagccctaccgtggcctactcggtcctggttgtggcgaaggttgagaagggcaagtccaagaagctcaagagcgtgaaggagctcctggggatcaccatcatggagaggtccagcttcgagaagaacccaatcgacttcctggaggccaagggctacaaggaggtgaagaaggacctgatcatcaagctcccgaagtactctctcttcgagctggagaacggcaggaagagaatgctggcttccgctggcgagctccagaaggggaacgagctcgcgctgccaagcaagtacgtgaacttcctctacctggcttcccactacgagaagctcaagggcagcccggaggacaacgagcaaaagcagctgttcgtcgagcagcacaagcattacctcgacgagatcatcgagcaaatctccgagttcagcaagcgcgtgatcctcgccgacgcgaacctggataaggtcctctccgcctacaacaagcaccgggacaagcccatcagagagcaagcggagaacatcatccatctcttcaccctgacgaacctcggcgctcctgctgctttcaagtacttcgacaccacgatcgatcggaagagatacacctccacgaaggaggtcctggacgcgaccctcatccaccagtcgatcaccggcctgtacgagacgaggatcgacctctcacaactcggcggggataagagacccgcagcaaccaagaaggcagggcaagcaaagaagaagaagacgcgt**ccaaagaagaagcggaaggtg

### GhABE7.10dCpf1

(NLS-TadA(wt)–linker(32 aa)–TadA*(7.10)–linker(32 aa)–dCpf1(D832A/E1006A/D1125A)–NLS

**ATG**ccaaagaagaagaggaaggtt**TCTGAAGTTGAGTTCTCACATGAATATTGGATGAGACATGCTCTTACATTGGCTAAGAGAGCTTGGGATGAAAGGGAGGTTCCTGTTGGTGCTGTTCTTGTTCATAATAACAGGGTTATTGGTGAAGGATGGAATAGACCTATCGGAAGGCATGATCCAACCGCTCATGCTGAGATTATGGCTCTTAGACAAGGTGGATTGGTTATGCAAAACTATAGGTTGATCGATGCTACACTTTACGTTACCTTGGAGCCTTGTGTTATGTGCGCTGGTGCTATGATTCATTCTAGAATCGGTAGGGTTGTTTTTGGAGCTAGAGATGCTAAGACCGGTGCTGCTGGATCACTTATGGATGTTTTGCATCATCCTGGTATGAATCATAGAGTTGAAATTACTGAGGGAATCCTTGCTGATGAATGTGCTGCTCTTTTGTCTGATTTCTTTAGGATGAGAAGGCAAGAGATTAAAGCTCAAAAGAAAGCTCAATCTTCAACAGAT**TCTGGTGGATCTTCAGGTGGATCTTCAGGTTCAGAAACTCCTGGAACATCTGAATCAGCTACCCCAGAGTCATCAGGTGGATCATCAGGTGGATCT**TCAGAGGTGGAGTTCTCACATGAGTACTGGATGAGGCACGCTCTTACTTTGGCTAAGAGAGCTAGAGACGAGAGAGAAGTGCCAGTGGGTGCTGTTCTTGTTTTGAATAACAGAGTTATTGGAGAGGGATGGAATAGGGCTATCGGACTTCATGATCCAACAGCCCACGCCGAGATTATGGCCTTGAGGCAAGGTGGATTGGTTATGCAGAACTATAGGCTTATCGATGCTACTCTTTATGTTACATTTGAACCATGCGTGATGTGTGCCGGAGCCATGATCCACTCAAGGATTGGAAGGGTTGTTTTTGGAGTTAGAAATGCTAAAACTGGAGCCGCCGGTTCTCTTATGGATGTGTTGCATTATCCTGGTATGAACCATAGGGTTGAAATTACAGAGGGAATCTTGGCCGACGAGTGCGCTGCTCTTTTGTGCTACTTCTTTAGAATGCCAAGACAAGTTTTTAACGCCCAAAAGAAAGCCCAATCTTCAACCGAC**TCTGGTGGATCATCAGGTGGAAGTTCAGGTTCAGAGACCCCTGGAACTTCTGAATCAGCTACTCCAGAGTCCTCAGGTGGATCATCAGGTGGATCG**TCTAAACTTGAGAAGTTTACCAACTGTTACTCTCTTTCAAAAACTTTGAGATTCAAGGCTATTCCTGTTGGAAAAACTCAAGAGAATATCGATAACAAGAGACTTTTGGTTGAAGATGAGAAAAGGGCTGAAGATTACAAGGGTGTTAAGAAACTTTTGGATAGGTACTACCTTTCTTTTATTAATGATGTTTTGCATTCAATCAAACTTAAGAACTTGAATAACTACATTTCTCTTTTCAGAAAGAAAACCAGGACTGAAAAAGAGAACAAGGAACTTGAGAATTTGGAGATTAACCTTAGAAAGGAAATCGCTAAAGCTTTCAAGGGTAACGAAGGATACAAATCATTGTTTAAGAAGGACATCATCGAGACCATCCTTCCAGAATTCTTGGATGATAAGGATGAGATCGCTCTTGTTAATTCTTTCAACGGTTTTACTACAGCTTTCACTGGATTCTTTGATAACAGGGAAAACATGTTCTCAGAAGAGGCTAAGTCTACTTCAATTGCTTTCAGATGCATCAATGAGAACTTGACAAGGTACATCTCTAACATGGATATCTTCGAAAAGGTTGATGCTATCTTCGATAAGCATGAAGTTCAAGAGATTAAAGAAAAGATCCTTAACTCAGATTACGATGTTGAGGATTTCTTTGAAGGAGAGTTCTTTAACTTCGTTTTGACACAAGAAGGAATCGATGTTTACAACGCTATTATCGGTGGATTCGTTACCGAGTCTGGTGAAAAAATTAAGGGACTTAACGAATATATCAATTTGTACAACCAAAAGACTAAGCAAAAGCTTCCTAAGTTTAAACCACTTTACAAGCAAGTTTTGTCTGATAGAGAGTCTTTGTCATTCTATGGTGAAGGATACACCTCAGATGAAGAGGTTCTTGAGGTTTTTAGGAACACTTTGAATAAGAACTCAGAAATTTTCTCTTCAATCAAGAAACTTGAGAAGTTGTTCAAGAACTTCGATGAATACTCTTCAGCTGGTATCTTCGTTAAAAATGGTCCTGCTATCTCTACAATCTCAAAGGATATCTTCGGAGAGTGGAATGTTATCAGGGATAAGTGGAACGCTGAATACGATGATATCCATCTTAAGAAAAAGGCTGTTGTTACCGAGAAATATGAAGATGATAGAAGGAAGTCTTTCAAAAAGATCGGATCTTTTTCACTTGAGCAATTGCAAGAATACGCTGATGCTGATCTTTCAGTTGTTGAGAAATTGAAGGAAATTATCATTCAAAAGGTTGATGAGATCTATAAGGTTTACGGTTCTTCAGAAAAATTGTTCGATGCTGATTTCGTTCTTGAGAAGTCTTTGAAAAAGAATGATGCTGTTGTTGCTATTATGAAAGATCTTTTGGATTCTGTTAAGTCATTCGAGAACTATATCAAAGCTTTCTTTGGAGAGGGAAAGGAAACTAACAGAGATGAATCTTTCTACGGAGATTTCGTTCTTGCTTACGATATCCTTTTGAAGGTTGATCATATCTATGATGCTATCAGGAACTACGTTACACAAAAGCCTTACTCAAAGGATAAATTCAAGTTGTACTTCCAAAACCCACAATTCATGGGTGGATGGGATAAAGATAAGGAAACCGATTACAGAGCTACTATCCTTAGATACGGTTCTAAGTATTACTTGGCTATCATGGATAAGAAGTACGCTAAGTGTCTTCAAAAGATTGATAAGGATGATGTTAATGGTAACTACGAGAAAATCAATTACAAGCTTTTGCCTGGACCAAACAAAATGTTGCCTAAGGTTTTCTTTTCTAAAAAGTGGATGGCTTACTACAACCCATCAGAAGATATCCAAAAGATCTATAAGAACGGTACATTCAAAAAGGGAGATATGTTCAATCTTAACGATTGCCATAAGTTGATCGATTTCTTTAAGGATTCTATCTCAAGATACCCTAAGTGGTCTAACGCTTACGATTTCAACTTCTCAGAAACCGAGAAGTACAAGGATATCGCTGGTTTCTACAGAGAGGTTGAAGAGCAAGGATACAAGGTTTCTTTCGAGTCTGCTTCAAAAAAGGAAGTTGATAAGCTTGTTGAAGAAGGAAAGCTTTACATGTTCCAAATCTATAATAAGGATTTTTCTGATAAGTCACATGGAACACCAAACCTTCATACCATGTACTTCAAGCTTTTGTTCGATGAGAATAACCATGGACAAATTAGGCTTTCTGGTGGAGCTGAATTGTTCATGAGAAGGGCTTCACTTAAAAAGGAAGAGTTGGTTGTTCATCCTGCTAATTCACCAATCGCTAATAAGAACCCTGATAACCCAAAAAAGACCACTACACTTTCTTACGATGTTTACAAAGATAAGAGATTTTCAGAGGATCAATACGAATTGCATATCCCTATCGCTATCAACAAATGTCCAAAGAACATTTTCAAAATCAACACTGAGGTTAGGGTTCTTTTGAAGCATGATGATAATCCTTACGTTATTGGTATCGCTAGAGGAGAAAGGAACCTTTTGTATATTGTTGTTGTTGATGGAAAGGGAAATATCGTTGAGCAATACTCTCTTAACGAAATTATTAATAACTTCAACGGTATCAGAATCAAGACTGATTACCATTCACTTTTGGATAAGAAGGAAAAGGAGAGATTCGAAGCTAGGCAAAATTGGACATCTATTGAGAACATCAAAGAACTTAAGGCTGGATACATCTCACAAGTTGTTCATAAGATCTGTGAGTTGGTTGAAAAGTATGATGCTGTTATTGCTCTTGCTGATTTGAATTCTGGTTTTAAGAACTCAAGGGTTAAAGTTGAGAAGCAAGTTTACCAAAAGTTCGAAAAGATGCTTATCGATAAGTTGAACTACATGGTTGATAAAAAGTCTAACCCATGCGCTACAGGTGGAGCTCTTAAGGGTTACCAAATCACCAACAAATTCGAGTCTTTCAAGTCTATGTCAACACAAAATGGTTTCATTTTCTATATCCCTGCTTGGTTGACTTCTAAGATTGATCCATCAACAGGTTTCGTTAACCTTTTGAAGACAAAGTACACCTCTATCGCTGATTCAAAAAAGTTCATTTCTTCATTCGATAGAATCATGTATGTTCCTGAAGAGGATCTTTTCGAATTCGCTTTGGATTACAAGAACTTCTCTAGGACCGATGCTGATTATATTAAAAAGTGGAAACTTTACTCATACGGAAACAGAATCAGGATCTTCAGAAACCCAAAAAAGAATAACGTTTTCGATTGGGAAGAGGTTTGTCTTACTTCTGCTTACAAAGAGTTGTTTAATAAGTACGGTATTAACTACCAACAAGGAGATATCAGGGCTCTTTTGTGCGAACAATCAGATAAGGCTTTCTATTCTTCTTTCATGGCTCTTATGTCTCTTATGTTGCAAATGAGAAACTCAATTACTGGTAGGACAGATGTTGCTTTCCTTATTTCTCCTGTTAAGAACTCAGATGGAATCTTCTATGATTCTAGAAACTACGAGGCTCAAGAAAACGCTATTCTTCCAAAGAATGCTGATGCTAACGGTGCTTACAATATTGCTAGAAAAGTTTTGTGGGCTATCGGACAATTCAAAAAGGCTGAAGATGAGAAGCTTGATAAGGTTAAGATCGCTATCTCAAACAAGGAGTGGTTGGAATATGCTCAAACTTCTGTTAAACATGGTTCAacgcgt**ccaaagaagaagcggaaggtg
